# Netazepide, a Gastrin Receptor Antagonist, Normalises Tumour Biomarkers and Causes Regression of Type 1 Gastric Neuroendocrine Tumours in a Nonrandomised Trial of Patients with Chronic Atrophic Gastritis

**DOI:** 10.1371/journal.pone.0076462

**Published:** 2013-10-01

**Authors:** Andrew R. Moore, Malcolm Boyce, Islay A. Steele, Fiona Campbell, Andrea Varro, D. Mark Pritchard

**Affiliations:** 1 Department of Gastroenterology, Institute of Translational Medicine, University of Liverpool, Liverpool, United Kingdom; 2 Department of Cellular and Molecular Physiology, Institute of Translational Medicine, University of Liverpool, Liverpool, United Kingdom; 3 Hammersmith Medicines Research, London, United Kingdom; 4 Department of Pathology, Royal Liverpool and Broadgreen University Hospitals NHS Trust, Liverpool, United Kingdom; Centro di Riferimento Oncologico, IRCCS National Cancer Institute, Italy

## Abstract

**Introduction:**

Autoimmune chronic atrophic gastritis (CAG) causes hypochlorhydria and hypergastrinaemia, which can lead to enterochromaffin-like (ECL) cell hyperplasia and gastric neuroendocrine tumours (type 1 gastric NETs). Most behave indolently, but some larger tumours metastasise. Antrectomy, which removes the source of the hypergastrinaemia, usually causes tumour regression. Non-clinical and healthy-subject studies have shown that netazepide (YF476) is a potent, highly selective and orally-active gastrin/CCK-2 receptor antagonist. Also, it is effective in animal models of ECL-cell tumours induced by hypergastrinaemia.

**Aim:**

To assess the effect of netazepide on tumour biomarkers, number and size in patients with type I gastric NETs.

**Methods:**

We studied 8 patients with multiple tumours and raised circulating gastrin and chromogranin A (CgA) concentrations in an open trial of oral netazepide for 12 weeks, with follow-up 12 weeks later. At 0, 6, 12 and 24 weeks, we carried out gastroscopy, counted and measured tumours, and took biopsies to assess abundances of several ECL-cell constituents. At 0, 3, 6, 9, 12 and 24 weeks, we measured circulating gastrin and CgA and assessed safety and tolerability.

**Results:**

Netazepide was safe and well tolerated. Abundances of CgA (p<0.05), histidine decarboxylase (p<0.05) and matrix metalloproteinase-7(p<0.10) were reduced at 6 and 12 weeks, but were raised again at follow-up. Likewise, plasma CgA was reduced at 3 weeks (p<0.01), remained so until 12 weeks, but was raised again at follow-up. Tumours were fewer and the size of the largest one was smaller (p<0.05) at 12 weeks, and remained so at follow-up. Serum gastrin was unaffected.

**Conclusion:**

The reduction in abundances, plasma CgA, and tumour number and size by netazepide show that type 1 NETs are gastrin-dependent tumours. Failure of netazepide to increase serum gastrin further is consistent with achlorhydria. Netazepide is a potential new treatment for type 1 NETs. Longer, controlled trials are justified.

**Trial Registration:**

European Union EudraCT database 2007-002916-24 https://www.clinicaltrialsregister.eu/ctr-search/search?query=2007-002916-24 ClinicalTrials.gov NCT01339169 http://clinicaltrials.gov/ct2/show/NCT01339169?term=yf476&rank=5

## Introduction

Patients with autoimmune chronic atrophic gastritis develop hypergastrinaemia secondary to hypochlorhydria. Hypergastrinaemia can lead to hyperplasia of enterochromaffin-like (ECL) cells in the gastric mucosa, which in some patients progresses to dysplasia and development of neuroendocrine tumours (NETs), known as type 1 gastric NETs [1,2]. They are often multiple, usually occur in the gastric corpus and fundus, are the commonest type of gastric neuroendocrine (carcinoid) tumour, and their incidence is increasing [3-5].

There are several management options for such patients. Tumours <1 cm diameter rarely metastasise, have a good prognosis, and are usually managed by endoscopic surveillance or resection [6-11]. However, endoscopic surveillance is burdensome for the patient, and endoscopic resection does not remove the source of the hypergastrinaemia and is difficult if there are numerous tumours. Tumours >1 cm diameter have the potential to metastasise, so additional treatment should be considered. One option is surgical antrectomy, which removes the anatomical source of hypergastrinaemia and reduces serum gastrin concentrations. Antrectomy can cause complete regression of type 1 gastric NETs, but not in all patients [12-17], and it carries the risks of surgery. Another option is a somatostatin analogue, such as octreotide, which reduces tumour number and size, reduces serum gastrin concentrations indirectly, and reduces circulating chromogranin A (CgA) [18-21], which is a marker of ECL-cell mass and activity [22-24]. Somatostatin analogues must be given by injection, and are not always well tolerated [25].

Thus, existing treatments for type 1 gastric NETs all have their disadvantages. A gastrin/CCK-2 receptor antagonist, to block the effects of hypergastrinaemia, would seem a more logical treatment, given that type 1 gastric NETs originate from ECL cells, which possess the gastrin/CCK-2 receptors through which gastrin mediates its trophic effect. Gastrin has dual effects: it stimulates gastric mucosal cell growth, especially of ECL cells, as well as gastric acid secretion [26].

Many gastrin/CCK-2 receptor antagonists have been described [27,28], but none has been developed as a medicine [29], mainly because of problems with potency, selectivity and oral bioavailability. In non-clinical studies, netazepide (YF476) is a potent, highly selective and competitive gastrin/CCK-2 receptor antagonist, and has good oral bioavailability [30-32]. Activity persists during repeated dosing [33]. Netazepide prevents [34,35] and causes regression [35] of ECL tumours induced by hypergastrinaemia in rodents, and is active in animal models of gastric cancer [36,37]. In healthy subjects, oral netazepide is well tolerated and causes dose-dependent and persistent inhibition of the response to pentagastrin [38-40], and abolishes the increase in plasma CgA induced by hypergastrinaemia secondary to gastric acid suppression by a proton pump inhibitor [41].

Thus, there are compelling reasons to test netazepide in patients with hypergastrinaemia, especially those with type 1 gastric NETs. Here we report a pilot trial of the first administration of netazepide to patients. Our objectives were to assess the safety, tolerability and efficacy of netazepide for 12 weeks in patients with autoimmune chronic atrophic gastritis and multiple type 1 gastric NETs. Twelve weeks was the maximum time permitted by the current toxicology studies in rat and dog [41]. We assessed efficacy by: counting the number of tumours; measuring the diameter of the largest tumour; measuring abundances of the gastrin-dependent genes, chromogranin A, histidine decarboxylase, matrix metalloproteinase-7, and plasminogen activator inhibitor-1 and -2 in tumour biopsies, all of which are increased in type 1 gastric NETs [42-45]; and measuring plasma CgA concentration. We assessed a range of potential tumour biomarkers, because there is no published evidence about the effect of a gastrin/CCK-2 receptor antagonist on gene expression in humans, and we wanted to prepare for future studies.

## Materials and Methods

The protocol for this trial and supporting TREND checklist are available as supporting information; see Protocol S1 and Checklist S1.

### Ethics

We complied with the Declaration of Helsinki and ICH Guideline for Good Clinical Practice. The Medicines and Healthcare products Regulatory Agency, UK, and Cambridge East Research Ethics Committee, UK, approved the study. Patients gave written, informed consent. The trial was registered before the start (EUDRaCT/2007-002916-24) and retrospectively, in April 2011 at (ClinicalTrials.gov/NCT01339169). We did it between November 2010 and July 2012.

### Patients

Patients were eligible if they attended regularly the Neuroendocrine Tumour Clinic, Royal Liverpool and Broadgreen University Hospitals, and were known to have autoimmune chronic atrophic gastritis, hypergastrinaemia, raised serum CgA, and multiple type I gastric NETs, and were otherwise in good health. Exclusion criteria included: previous gastric surgery; treatment with somatostatin analogues; Zollinger-Ellison syndrome; prolonged QTc interval; and pregnancy, lactation or steroid contraceptive use in females.

### Study design

The trial was a single-centre, open-label, exploratory, phase 2 trial that was nonrandomised in design (Table 1). Patients entered the trial consecutively as they were recruited from the Neuroendocrine Centre. Trio Medicines Ltd, London, England supplied netazepide 25 mg capsules. Patients took netazepide 50 mg daily by mouth after breakfast for 12 weeks, and were followed up 12 weeks later. They visited the clinic 6 times. At 0, 3, 6, 9, 12 and 24 weeks, we collected blood for assay of fasting serum gastrin and plasma CgA concentrations, and assessed safety and tolerability by vital signs, ECG, safety tests of blood and urine, and adverse events. At 0, 6, 12 and 24 weeks, we did a gastroscopy, and at 3, 6, 9 and 12 weeks, we collected blood before and 1 hour after dosing, for assay of fasting plasma netazepide. Patients recorded when they took netazepide and any adverse events or concomitant medications, in a daily diary card. They returned their completed diary card and the used container of netazepide at subsequent visits, when we reviewed their diary entries and counted their remaining capsules, to assess tolerability and treatment compliance.

**Table 1:** Study design.

**Procedure**	**Clinic visit at week:**					
	**0^1^**	**3**	**6**	**9**	**12**	**24^2^**
Gastroscopy and biopsies	•		•		•	•
Real-time PCR abundances	•		•		•	•
Plasma CgA assay	•	•	•	•	•	•
Serum gastrin assay	•	•	•	•	•	•
Netazepide assay		•	•	•	•	
Safety and tolerability	•	•	•	•	•	•
Diary card	•	•	•	•	•	•

1 Start of 12 weeks’ netazepide 50 mg once daily

2 Follow-up, 12 weeks after end of netazepide treatment

### Endoscopy

The same endoscopist (DMP) and assistant (ARM) performed all the gastroscopies, using an Olympus GIF-Q series flexible video endoscope, in the endoscopy unit, Royal Liverpool and Broadgreen University Hospitals. Visible tumours were photographed, counted, and the diameter of the largest one estimated by comparison with an opened pair (9 mm) of biopsy forceps (Radial Jaw^TM^ 4, Boston Scientific, MA, USA). Mucosal pinch biopsies were taken from gastric antrum and corpus and from tumours, for routine histopathology (4 per site). Eight additional biopsies were taken from the gastric corpus and stored in RNAlater (Ambion, Austin, TX, USA), for subsequent assessment of real-time real-time polymerase-chain reaction (PCR) abundances of biomarkers.

### Histopathology and immunohistochemistry

We fixed biopsy samples in 10% neutral-buffered formalin and embedded them in paraffin before staining with hematoxylin and eosin. We also processed biopsy samples for immunohistochemical detection of synaptophysin, Ki67 and CgA, using monoclonal mouse antihuman synaptophysin antibody at 1:80 (NCLSynap299, Leica Microsystems Inc. IL, USA), monoclonal mouse antihuman Ki67 antibody at 1:200 (NCL-Ki67-MM1, Leica Microsystems Inc. IL, USA) and polyclonal rabbit antihuman CgA antibody at 1:8000 (A0430, Dako, Denmark), respectively. The same expert gastrointestinal histopathologist (FC) examined specimens and reported findings [46].

### Plasma chromogranin A

We collected blood (4 ml) in EDTA tubes, separated plasma by centrifugation, and stored it at -80°C until assay for CgA by Hammersmith Medicines Research, Cumberland Avenue, London (HMR), by ELISA (Kit K0025, DAKO, Denmark). Normal range and coefficient of variation are 2–22 U/L (7.2%).

### Serum gastrin

We collected venous blood (2.5 ml) in serum tubes, allowed it to clot at room temperature for at least 20 min, separated serum by centrifugation (4°C, 1500 G for 10 min) and stored it at -20°C until assay for amidated gastrin concentrations by two methods: radioimmunoassay and ELISA. For the radioimmunoassays (RIA), we used antibody L2, which reacts at the COOH terminus of G17 and measures G17, G34, and minor components, such as G14 with similar affinity [47,48], and for the ELISA, we used a commercial kit, Immunolite 2000, DPC (Siemens Healthcare Diagnostics Inc., NY, USA). Normal ranges and coefficient of variation of the two methods are <40 pM (10.7%) and 6–56 pmol/L (6.9%), respectively.

### Gastric mucosal biomarkers

We stored gastric corpus biopsies in RNAlater at -20°C before RNA extraction in Tri-Reagent (Sigma-Aldrich Company Ltd, Dorset, UK) according to the manufacturer’s instructions. We did real-time PCR using TaqMan chemistry with Precision 2× master mix (Primer Design Ltd, Southampton, UK) and a 7500 real-time PCR system (Applied Biosystems, Warrington, UK), as previously reported42–45. We assessed histidine decarboxylase (HDC), CgA, matrix metalloproteinase (MMP)-7, plasminogen activator inhibitor (PAI)-1 and 2 abundances relative to glyceraldehyde 3-phosphate dehydrogenase (GAPDH). See Table 2 for primers and probe sets.

**Table 2:** Primer and probe sets used for real-time PCR tests.

**GAPDH**		
Probe	hGAPDH-T71	CGT CGC CAG CCG AGC CAC A
Forward Primer	hGAPDH-F34	GCT CCT CCT GTT CGA CAG TCA
Reverse Primer	hGAPDH-R113	ACC TTC CCC ATG GTG TCT GA
**CgA**		
Probe	hCgA-T400	CCA GCC CCA TGC CTG TCA GCC
Forward Primer	hCgA-F345	GAT ACC GAG GTG ATG AAA TGC A
Reverse Primer	hCgA-R493	TCC TTC AGT AAA TTC TGA TGT CTC AGA
**HDC**		
Probe	hHDC-R91	CTC TGT TAA ACT CTG GTT CGT GAT TCG GTC C
Forward Primer	hHDC-F91	CCC TGA GCC GAC GGT TT
Reverse Primer	hHDC-R91	GTA CCA TGT CTG ACA TGT GCT TGA
**MMP-7**		
Probe	hMMP-7-T651	CCT GTA TGC TGC AAC TCA TGA ACT TGG C
Forward Primer	hMMP-7-F624	GGA TGG TAG CAG TCT AGG GAT TAA CT
Reverse Primer	hMMP-7-R702	GGA ATG TCC CAT ACC CAA AGA A
**PAI-1**		
Probe	hPAI-1-T770	AGT TCA ACT ATA CTG AGT TCA CCA CGC CCG
Forward Primer	hPAI-1-F746	TGC CCA TGA TGG CTC AGA
Reverse Primer	hPAI-1-R829	GCA GTT CCA GGA TGT CGT AGT AAT G
**PAI-2**		
Probe	hPAI-2-T266	CCA ATG CAG TTA CCC CCA TGA CTC CA
Forward Primer	hPAI-2-F234	GGC CAA GGT GCT TCA GTT TAA T
Reverse Primer	hPAI-2-R316	TGA ACC CAC AGC TGG TAA AGT TC

### Plasma netazepide

We collected venous blood into lithium-heparin tubes, separated plasma by centrifugation (4°C, 1500 G for 10 min), and stored it at -20°C until assay of netazepide by Advisory Services Ltd, St George’s Hospital, London, UK, by HPLC/MS [49].

### Power

The study was exploratory, so we did not do a power calculation. Our hypothesis was that, by blocking gastrin/CCK-2 receptors on ECL cells, netazepide would reduce the number and size of type 1 gastric NETs, and perhaps even eradicate them, similar to antrectomy [12-17], and would reduce the real-time PCR abundances of the tumour biomarkers and plasma CgA. Therefore, we considered 8 patients enough to show a meaningful response to netazepide. We expected serum gastrin concentration to increase further only if patients still had functioning parietal cells for netazepide to suppress any residual gastric acid production.

### Statistical analysis

We analysed the outcome measures by the nonparametric Wilcoxon signed-rank test using SPSS version 20 as not all the data were normally distributed, and accepted p<0.05 as significant.

## Results

### Patient characteristics

We enrolled 8 patients (4 women and 4 men; mean age 66 years, range 55–76 years) consecutively over about 12 months (Table 3). All 8 patients completed the study without deviating from the protocol. All patients had been investigated before and shown to have histologically confirmed type I gastric NETs and vitamin B12 deficiency. Two had anti-intrinsic factor antibodies, and all had anti-parietal cell antibodies. No patient had current *H. pylori* infection at routine histopathology, including immunohistochemistry, although two had *H. pylori* antibodies. No patient had evidence of metastases at computed tomography or somatostatin-receptor scintigraphy by ^111^In-octreotide scan.

**Table 3:** Patient characteristics.

Patient number	1	2	3	4	5	6	7	8
Age	60	64	67	69	76	67	55	66
Gender	Female	Female	Male	Male	Female	Female	Male	Male
Number of gastric polyps	8	8	4	9	30	10	12	10
Size of largest polyp (mm)	6	15	3	5	7	8	10	10
Histology of tumour	Low grade NET	Low grade NET	ECL-M	Low grade NET	Low grade NET	Low grade NET	Low grade NET	Low grade NET
Gastric corpus histology	AG, IM, ECL-M	AG, IM, ECL-M	AG, IM, ECL-L	AG, IM, ECL-M	AG, IM, low grade NET	AG, IM, ECL-M	AG, IM, ECL-D	AG, IM, ECL-D
Serum gastrin by RIA (pmol/L)	800	800	580	960	1050	470	1750	520
Serum gastrin by ELISA (pmol/L)	531	494	414	645	655	332	953	415
Serum CgA (U/L)	25.2	52.6	54	33	93	56	128	64
*H. pylori* histology	Negative	Negative	Negative	Negative	Negative	Negative	Negative	Negative
*H. pylori* serology	Negative	Negative	Negative	Not done	Negative	Positive	Negative	Positive
Vitamin B12 deficiency	Yes	Yes	Yes	Yes	Yes	Yes	Yes	Yes
Anti-parietal cell antibody	Positive	Positive	Positive	Positive	Positive	Positive	Positive	Positive
Anti-intrinsic factor antibody	Negative	Positive	Positive	Negative	Negative	Negative	Negative	Negative

AG = atrophic gastritis, IM = intestinal metaplasia, ECL-L = linear ECL-cell hyperplasia, ECL-M = micronodular ECL-cell hyperplasia, ECL-D = ECL-cell dysplasia, NET = neuroendocrine tumour.

### Tumour number and size

**Figure 1:**
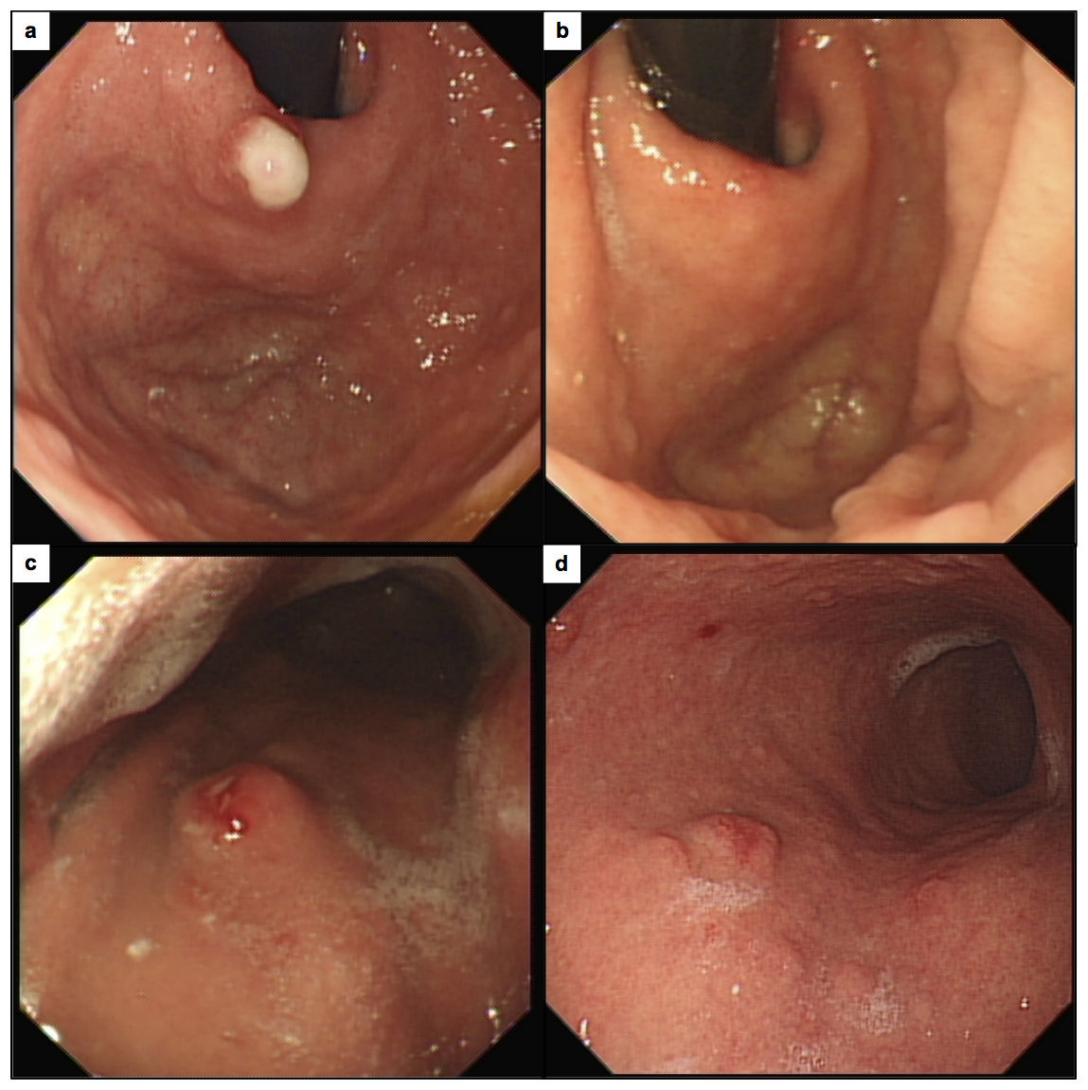
Endoscopic photographs from the same area of the stomach in patients 1 (a, b) and 2 (c, d) at baseline (a, c) and after 12 weeks of netazepide (b, d).

At baseline gastroscopy, all patients had visible gastric tumours (Figure 1). The median number was 10 (range 4–30), and the mean diameter of the largest was 6.75 mm (range 3–15 mm).

**Figure 2:**
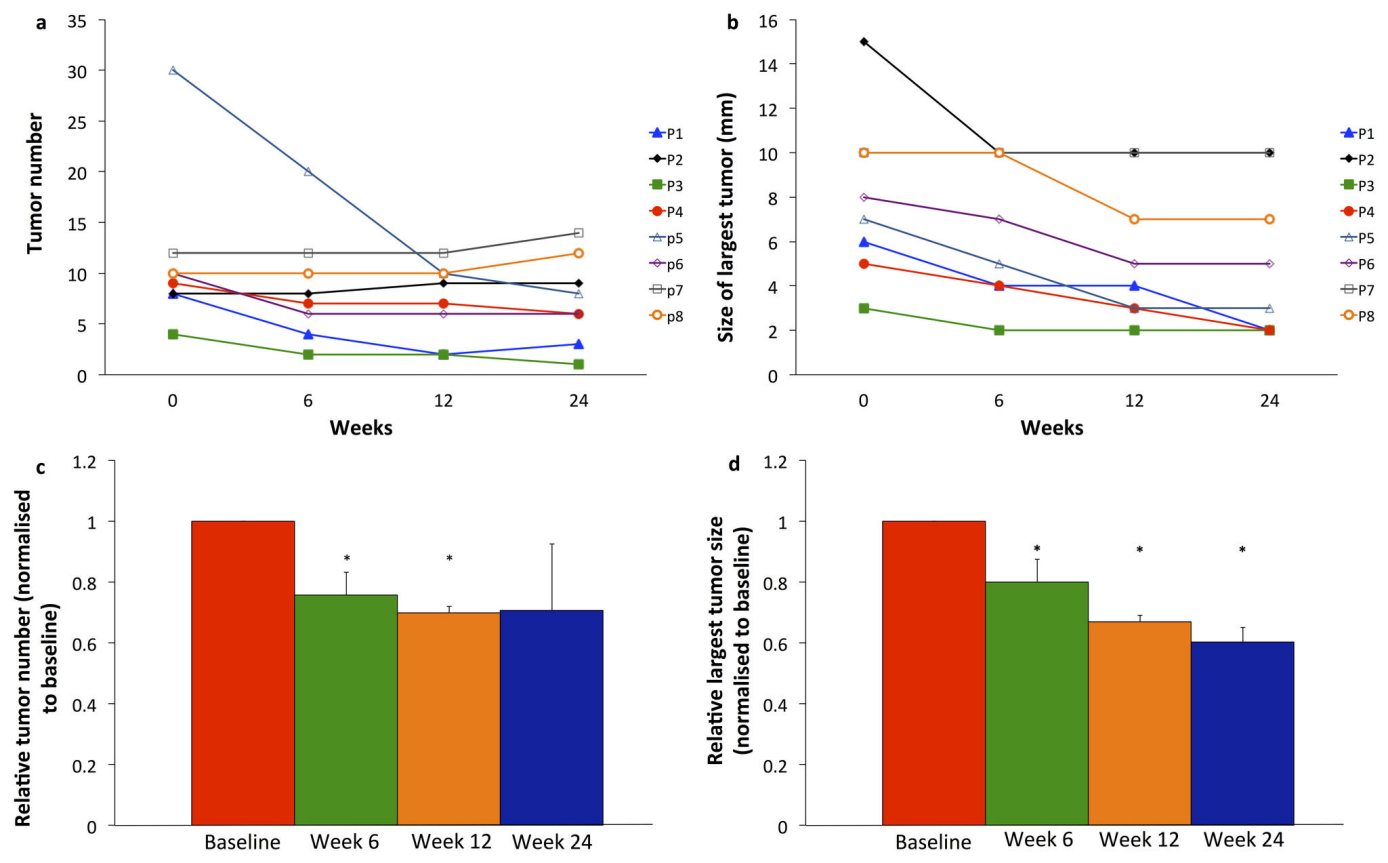
Endoscopic tumour characteristics: (a) number of tumours; (b) size of largest tumour, and (c,d) % change from baseline after 6 and 12 weeks’ netazepide treatment, and at follow-up at 24 weeks, 12 weeks after end of treatment.

In 5 patients, there were fewer tumours after 6 and/or 12 weeks’ treatment (Figure 2a). Of the other 3 patients, 2 had the same number and the other had a few more. After 6 and 12 weeks’ treatment, the mean decrease in the number of tumours relative to baseline was 24% (p=0.041) and 30% (p=0.046), respectively. At 24 weeks, 12 weeks after completion of treatment, findings were similar to those at 12 weeks. The mean decrease relative to baseline was 29% (p=0.092).

All but one patient had a decrease in diameter of their largest tumour after 6 and/or 12 weeks’ treatment (Figure 2b). The mean decrease relative to baseline was 20% (p=0.017) and 33% (p= 0.018), respectively. None of the largest tumours had increased in size at 12 weeks after stopping treatment, and 2 of them were slightly smaller. The mean decrease relative to baseline was 40% (p=0.017). Summary statistics for tumour characteristics can be found in Table S1.

### Histopathology

**Figure 3:**
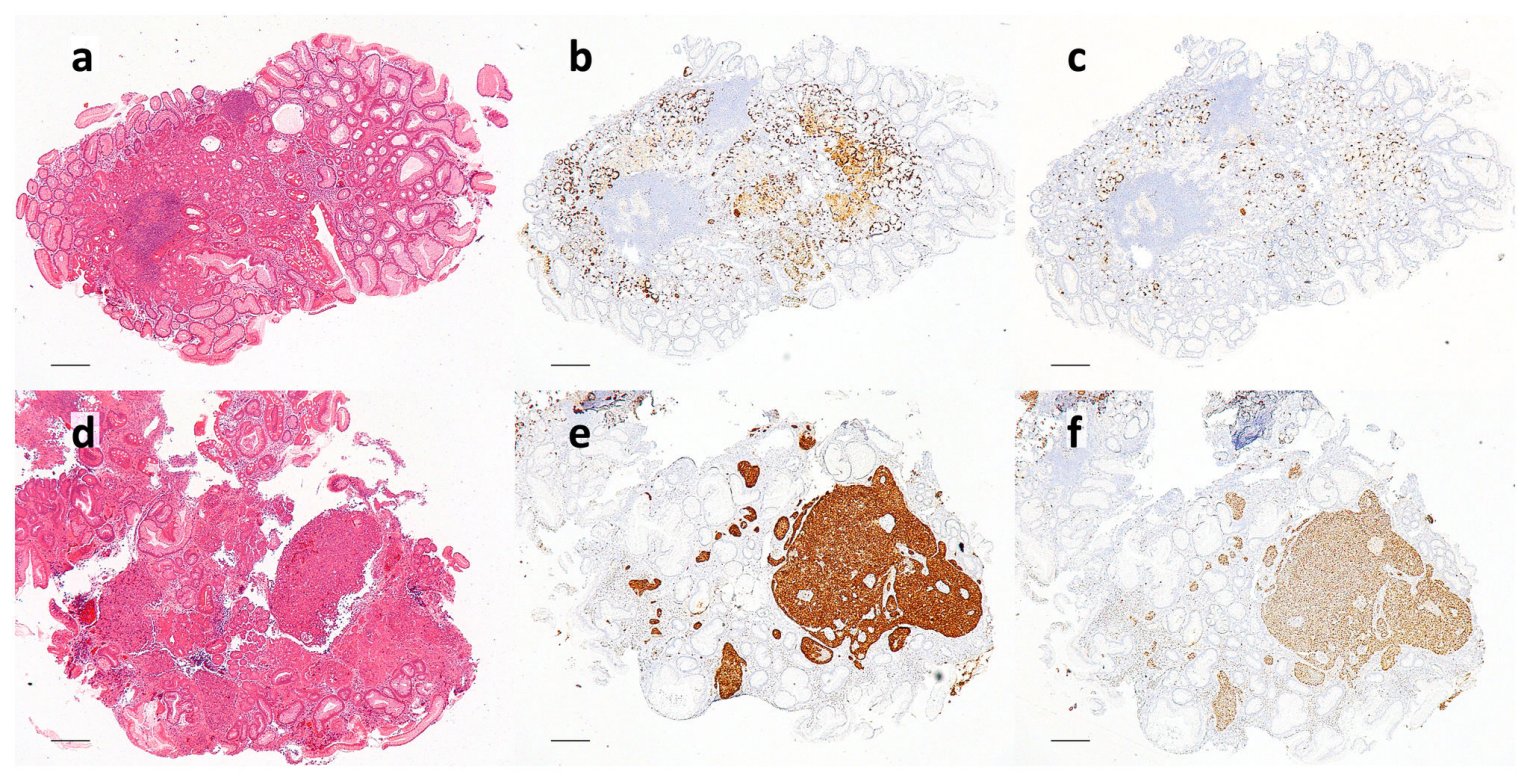
Representative photomicrographs of gastric corpus (a-c) and a neuroendocrine tumour (d-f) from the same patient stained for H and E (a,d), chromogranin A (b.e) and synaptophysin (c,f). Scale bar = 200µm.

All 8 patients had low-grade type 1 gastric NETs prior to enrolment. Seven still had low-grade NETs at baseline; the other had micronodular ECL-cell hyperplasia (ECL-M) throughout the study. He had a histologically confirmed small NET prior to enrolment, which presumably was removed by biopsy forceps at a previous endoscopy. All gastric corpus mucosal biopsies showed ECL-cell hyperplasia throughout the study, but there were no further morphological or histopathological changes. Examples of immunohistochemical stains are shown in Figure 3.

### Plasma chromogranin A and serum gastrin

**Figure 4:**
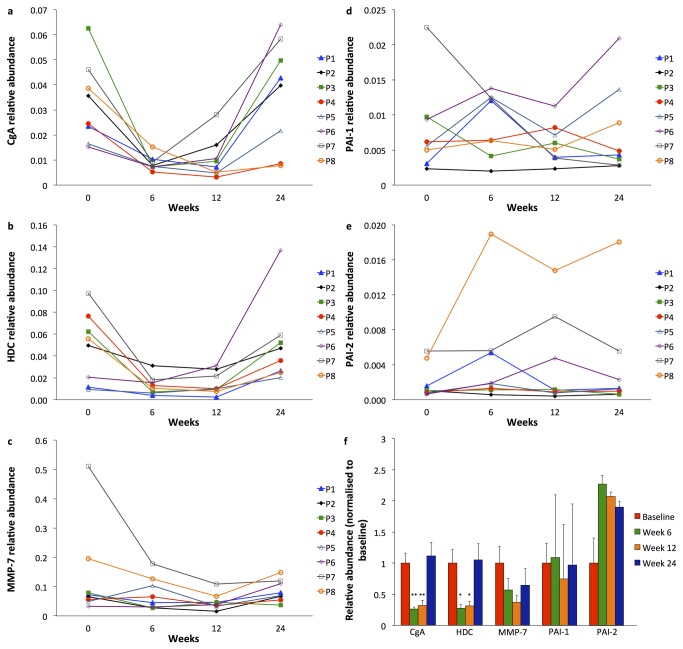
Fasting (a) plasma chromogranin A (U/L) and (b) serum gastrin (pmol/L) concentrations at baseline, after 3, 6, 9 and 12 weeks’ netazepide treatment, and at follow-up at 24 weeks, 12 weeks after end of treatment.

CgA and gastrin concentrations at baseline, 3, 6, 9 and 12 weeks and at follow-up at 24 weeks are shown in Figure 4.

After 3 weeks of netazepide, plasma CgA had decreased in all subjects (Figure 4a); mean decrease relative to baseline was 30% (p=0.012). The response was sustained throughout treatment; mean decrease at 12 weeks relative to baseline was 31% (p=0.012). Patient 5 appeared to respond less favourably; her diary card entries and capsule counts showed erratic treatment compliance. At follow-up, 12 weeks after treatment cessation, plasma CgA was raised again in all patients (mean 82% relative to baseline).

All patients had high serum gastrin concentrations at baseline; mean (range) was 866 pM (470–1750 pmol) by RIA, and 555 pM (332–953 pmol) by ELISA (Table 3). There were no significant changes during treatment (Figure 4b). Summary statistics for plasma CgA and serum gastrin concentrations can be found in Table S1.

### Gastric mucosal biomarkers

**Figure 5:**
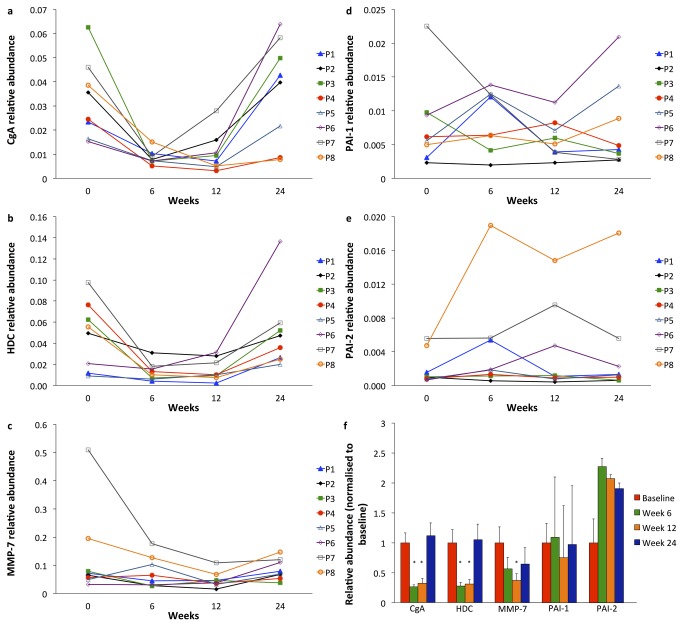
Gastric corpus mucosal mRNA abundance of CgA (a), HDC (b), MMP-7 (c), PAI-1 (d) and PAI-2 (e) normalised to mRNA abundance of the housekeeper gene GAPDH. Mean ± standard deviation of each biomarker after 6 and 12 weeks of netazepide treatment, and at follow-up at 24 weeks, 12 weeks after end of treatment (f). *p<0.05.

Real-time PCR abundances of the ECL-cell constituents CgA and HDC, normalised for the housekeeper gene GAPDH, decreased relative to baseline in all patients during netazepide treatment, and increased again after treatment cessation (Figure 5a and 5b, respectively). Mean real-time PCR abundance of CgA relative to baseline was 31% at 6 weeks (p=0.012) and 35% at 12 weeks (p=0.012). At 24 weeks, it was 138% of baseline (p=0.78). Mean real-time PCR abundance of HDC relative to baseline was 38% at 6 weeks (p=0.027) and 59% at 12 weeks (p=0.034). At follow-up, it was 179% relative to baseline (p=0.67). Mean real-time PCR abundance of MMP-7 relative to baseline was 82% at 6 weeks (p=0.16) and 56% at 12 weeks (p=0.017) (Figure 5c). At follow-up, it was 116% relative to baseline (p=0.78). The real-time PCR abundances of PAI-1 and PAI-2 did not change significantly (Figure 5d and 5e, respectively). Summary statistics for mucosal biomarker abundances can be found in Table S2.

### Pharmacokinetics

Plasma netazepide concentrations were measured before and 1 hour after dosing at 3, 6, 9 and 12 weeks (Table 4). Although there was a possible decrease in mean peak plasma concentration at the 12 week timepoint, this was not statistically significant.

**Table 4:** Mean ± standard deviation (SD) serum netazepide concentrations before (trough) and 1 hour (peak) after dosing at 3, 6, 9 and 12 weeks of treatment.

**Visit**	**Time**	**Mean concentration (ng/mL)**	**SD**
**3 weeks**	Trough	4.6	5.0
	Peak	132.4	183.5
**6 weeks**	Trough	7.0	4.4
	Peak	222.1	193.1
**9 weeks**	Trough	6.1	6.7
	Peak	151.3	214.5
**12 weeks**	Trough	5.9	4.4
	Peak	87.0	97.4

### Safety, tolerability and compliance

There were no adverse events that could be reasonably attributed to netazepide, and no clinically relevant changes in safety assessments. There was no evidence of a drug-drug interaction in those patients taking concomitant medication. Treatment compliance based on capsule counts and diary cards was 94% (SD 12%).

## Discussion

Netazepide was safe and well tolerated. Treatment compliance was high, consistent with good tolerability. The results provide good evidence of the efficacy of netazepide in patients with type 1 gastric NETs for several reasons.

First, there was a significant reduction in both the number of tumours and the size of the largest tumour, by about 30%, during netazepide treatment. We strove to minimise observer bias by having the same operator do all the gastroscopies, by taking multiple photographs of the gastric mucosa, and by identifying the largest tumour at baseline and measuring its diameter throughout the study by comparison with the open jaws of the adjacent biopsy forceps. We did not perform endoscopic ultrasound in this initial exploratory study as this would have significantly increased the procedure time and patients were not anaesthetised. However, the number of tumours is a better measure of efficacy than the size of the largest tumour, which is more subjective and lacks sensitivity for detecting small changes.

Second, there was a significant reduction in plasma CgA concentrations during netazepide treatment. Circulating CgA is a well-recognised marker of ECL-cell mass and activity [22-24]. The source of the CgA in CAG patients is either the ECL-cell hyperplasia/dysplasia in the gastric corpus mucosa or the ECL-cell tumours or both. CAG patients with or without ECL-cell tumours have equally raised concentrations of circulating CgA [50].

Third, there were significant reductions in real-time PCR abundances of CgA, HDC and MMP-7 in the gastric corpus mucosa. Intravenous octreotide gave a similar result in patients with type 1 gastric NETs, probably by reducing serum gastrin concentrations [51]. The reduction in real-time PCR abundance of CgA during netazepide therapy was not accompanied by a reduction in CgA-positive ECL cells in the gastric corpus mucosa, which mirrors our previous finding that ECL-cell hyperplasia persists in patients with type I gastric NETs after antrectomy even though there is a reduction in real-time PCR abundance of HDC [52]. We have previously shown that real-time PCR abundances of CgA, HDC, MMP-7, PAI-1 and PAI-2 are all increased in patients with CAG and hypergastrinaemia [42-45]. Therefore, we assessed a range of potential biomarkers, because there is no published evidence about the effect of a gastrin/CCK-2 receptor antagonist on gene expression in humans, and we wanted to prepare for future studies.

Fourth, there was a long follow-up period of 12 weeks, to assess reversal of any changes after stopping treatment. At follow-up, plasma CgA and the real-time PCR abundances of CgA, HDC and MMP-7 had returned to about pre-treatment levels. However, the reductions in the number of tumours and the diameter of the largest tumour during netazepide treatment persisted, probably because the tumours would have required much longer than 12 weeks of re-exposure to the trophic effect of hypergastrinaemia to regrow.

Finally, plasma concentrations of netazepide in CAG patients were similar to those in healthy subjects [38,39]. Such concentrations cause substantial antagonism of gastrin/CCK-2 receptor-mediated responses in healthy subjects. Despite having achlorhydria, CAG patients appear to absorb netazepide similarly to healthy subjects, which is reassuring given that hypoacidity induced by a proton pump inhibitor in healthy subjects impairs the bioavailability of some medicines [53,54].

Although serum gastrin concentrations by ELISA were lower than those by RIA, probably because the commercial kit that we used measures only a single gastrin form [55,56], both sets of results followed the same pattern. Netazepide did not affect serum gastrin in CAG patients, whereas netazepide increases it in rodents [33-37] and healthy subjects [39], secondary to suppression of gastric acid secretion. That netazepide did not further increase serum gastrin in our CAG patients is consistent with their having no functioning parietal cells. In other words, they had achlorhydria and no residual gastric acid production for netazepide to suppress. That serum gastrin was unchanged also emphasises that the effect of netazepide on type 1 gastric NETs is not indirect via suppression of serum gastrin.

Although no CAG patient had complete tumour regression, all but one patient had fewer and smaller tumours after 12 weeks’ netazepide treatment, and all had other evidence of a treatment effect. It can take at least 6 months after antrectomy for type 1 gastric NETS to regress completely in some patients [12-17], so it is not surprising that 12 weeks’ netazepide treatment failed to do so. Twelve weeks was the longest permitted by the regulatory authority on the basis of available non-clinical toxicology studies in rat and dog [41]. Longer-term treatment of patients will require toxicology studies for 6 and 9 months in rat and dog, respectively. Even if netazepide can eradicate type 1 gastric NETs, maintenance netazepide will probably be required to prevent recurrence.

There were limitations in the study design: it was open-label, lacked controls and the number of patients was small; duration of netazepide treatment was short; and there was a possibility of observer bias by the endoscopist. We decided upon an open, uncontrolled study design for several reasons. First, we took measures to minimise observer bias by the endoscopist. Second, plasma CgA and real-time PCR abundances are valid outcome measures. Third, type 1 gastric NETs are rare [1,3], and we wanted to offer all patients the possibility of active treatment. Fourth, the study was the first administration of netazepide to patients, and a ‘proof-of-principle’ exploratory study. Overall, the findings – clinical and laboratory – have face validity and provide good initial evidence of the effectiveness of netazepide in the treatment of type 1 gastric NETs. That conclusion is strengthened by a subsequent study in patients with multiple type 1 gastric NETs, which also showed that netazepide reduces tumour number and size and normalises serum CgA [57]. That study, unlike ours, did not report the effects of netazepide on tumour biomarkers.

Netazepide has been designated an orphan medicinal product for treatment of gastric NETs in Europe [58] and the USA [59].

## Conclusions

The reductions in abundances, circulating CgA, and tumour number and size by netazepide, a gastrin receptor antagonist, show that type 1 gastric NETS are gastrin-dependent tumours. Netazepide is a well-tolerated and a potential new targeted medical treatment for type 1 gastric NETs, and has advantages over existing treatments. Randomised, controlled trials of longer-term treatment in larger numbers of patients using similar outcome measures are justified.

## Supporting Information

Checklist S1 **TREND Checklist.**
(PDF)

Protocol S1 **Trial Protocol.**
(PDF)

Table S1 **Circulating biomarkers and endoscopic features.**
(DOCX)

Table S2 **Mucosal biomarkers.**
(DOCX)
