# Correction: Netazepide, a Gastrin Receptor Antagonist, Normalises Tumour Biomarkers and Causes Regression of Type 1 Gastric Neuroendocrine Tumours in a Nonrandomised Trial of Patients with Chronic Atrophic Gastritis

**DOI:** 10.1371/annotation/4afd48b1-8dc0-47cc-9c8a-99aba7623116

**Published:** 2013-11-01

**Authors:** Andrew R. Moore, Malcolm Boyce, Islay A. Steele, Fiona Campbell, Andrea Varro, D. Mark Pritchard

As a result of an error in the production process, a duplicate of Figure 5 appears in place of Figure 4.

The correct version of Figure 4 is available here: 

**Figure:**
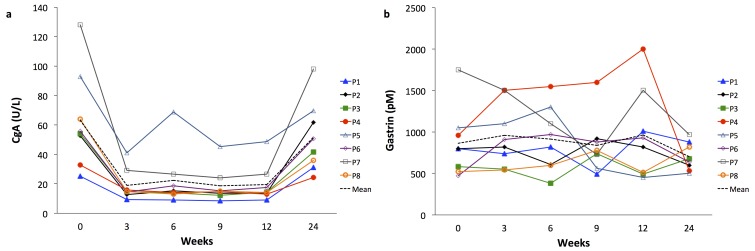


The legend for Figure 4 is correct. 

