# Supplementary material for: Netazepide, a Gastrin Receptor Antagonist, Normalises Tumour Biomarkers and Causes Regression of Type 1 Gastric Neuroendocrine Tumours in a Nonrandomised Trial of Patients with Chronic Atrophic Gastritis
Source: PLoS One. 2013 Oct 1;8(10):e76462. doi: 10.1371/journal.pone.0076462 (PMC3788129; doi:10.1371/journal.pone.0076462)
Supplement: Protocol S1 — Trial Protocol. (PDF) [file pone.0076462.s002.pdf]

TRIO

Trio Medicines Limited

Trial protocol

Confidential

**Trial title**	A single centre, pilot trial of YF476 in patients with chronic atrophic gastritis, hypergastrinaemia and type I gastric carcinoids.
**Short title**	YF476 and type I gastric carcinoids
**Protocol Version**	13, including protocol amendments 1–6
**Protocol Date**	26 February 2013
**Sponsor code**	T-008
**HMR code**	07-504
**EudraCT no**	2007-002916-24
**Trial medication**	YF476
**Phase of trial**	Phase 2
**Place of trial**	UK single-centre trial<br>Royal Liverpool University Hospital
**Principal investigator**	Professor Mark Pritchard<br>Royal Liverpool University Hospital
**Sponsor**	Trio Medicines Ltd<br>PO Box 53346<br>London NW10 7XU<br>UK
**Planned dates of trial**	July 2010 – January 2014

1 Signatures

The investigator and the sponsor have discussed this protocol. The investigator agrees to carry out the trial and to abide by this protocol, except in a medical emergency or when departures from it are mutually agreed in writing and meet the requirements of the regulatory authority and the ethics committee.

Principal investigator

Professor Mark Pritchard
Royal Liverpool University Hospital

Signature Date

Sponsor's medical monitor

Dr Malcolm Boyce
Trio Medicines Ltd

MJBoyce 26 FEB 13

Signature Date

Clinical Project Manager

Dr Gillian Turnbull
HMR

GTurnbull 26 FEB 2013

Signature Date

Statistician

Mrs Toni Mitchell
HMR

TRMitchell 28 FEB 2013

Signature Date

1 Signatures

The investigator and the sponsor have discussed this protocol. The investigator agrees to carry out the trial and to abide by this protocol, except in a medical emergency or when departures from it are mutually agreed in writing and meet the requirements of the regulatory authority and the ethics committee.

Principal investigator

Professor Mark Pritchard [illegible] 28 FEB 2013
Royal Liverpool University Hospital Signature Date

Sponsor's medical monitor

Dr Malcolm Boyce
Trio Medicines Ltd Signature Date

Clinical Project Manager

Dr Gillian Turnbull
HMR Signature Date

Statistician

Mrs Toni Mitchell
HMR Signature Date

2 Summary

2.1 Background

Chronic atrophic gastritis is characterised by gastric mucosal atrophy and achlorhydria. Achlorhydria stimulates hyperplasia of gastric G-cells, which results in hypergastrinaemia. Hypergastrinaemia causes ECL-cell hyperplasia which leads to growth of gastric tumours (type I carcinoids) in about 5% of patients. About 80% of all gastric carcinoids are type I. Gastric carcinoids are rare; the prevalence is <1 per 10,000 population. Although most type I gastric carcinoids are benign, they all have the potential to become malignant and metastasise. Gastric surgery is the usual treatment for troublesome carcinoids.

There is much evidence that type I carcinoids are gastrin dependent. Therefore, YF476 – a potent, orally active, highly selective, competitive antagonist of gastrin receptors – might obviate the need for surgical removal of gastric carcinoids. Indeed, the European Commission and the USA Food and Drug Administration (FDA) have designated YF476 an Orphan Medicinal Product for treatment of gastric carcinoids.

2.2 Trial medication

YF476 (50 mg: 2 x 25 mg capsules) by mouth *once* daily for 12 weeks. Taken with breakfast except on clinic visit days when patients will fast overnight and take YF476 after completion of assessments.

2.3 Objectives

The primary objective is:

- to assess if YF476 is an effective medical treatment for type I gastric carcinoids.

The secondary objectives are:

- to assess the tolerability and safety of YF476; and
- to assess the effect of YF476 on plasma concentration and transcript profiles of biomarkers such as chromogranin A (CgA).

2.4 Type of trial

Single centre, open-label, pilot, phase 2, out-patient trial.

2.5 Trial population

Total up to 10 patients.

Inclusion criteria

1. Patients known to have gastric carcinoids associated with chronic atrophic gastritis and hypergastrinaemia and who attend the out-patient clinic of the investigators.
2. Men; post-menopausal women; pre-menopausal women who have been sterilised by tubal ligation, hysterectomy or bilateral oophorectomy; or pre-menopausal women using one of the allowed methods of contraception: condom and spermicide or intra-uterine device.
3. Adults ≥18 years.
4. Good general health.
5. Able to give fully-informed, written consent.

Exclusion criteria

1. Women who are pregnant, lactating or using a steroid contraceptive.
2. History of gastric surgery, apart from surgery for gastric carcinoids.
3. Evidence of Zollinger-Ellison syndrome.
4. Prolonged QTc interval (> 450 msec).
5. Certain medicines and herbal remedies (see Appendix 1) taken during the 7 days before Visit 1.
6. Previous treatment with somatostatin analogues.
7. Participation in other clinical trials of unlicensed medicines within the previous 3 months.

2.6 Trial design

Patients will attend the clinic up to 12 times. The time that patients take to complete the trial will depend on their response to YF476.

At **Visit 1**, patients will be screened (excluding gastroscopy) for eligibility.

At **Visit 2**, patients will undergo gastroscopy and gastric biopsies. If they meet all the inclusion/exclusion criteria, they will start treatment with 50 mg YF476 by mouth *once* daily at home on the next day. Each subject will be given a unique number at the start of treatment.

They will complete a diary card daily throughout the treatment period.

Visit 3 will be 3 weeks after starting YF476, to assess tolerability, safety and blood levels of gastrin, CgA and YF476.

Visit 4 will be 6 weeks after starting YF476, to assess tolerability and safety, to repeat the gastroscopy and gastric biopsies, and to take blood samples to assess levels of gastrin, CgA and YF476. If the gastric carcinoids have regressed completely on visual inspection, YF476 will be stopped, Visits 5 and 6 will be

omitted, and the patient will proceed to Visit 7. If the gastric carcinoids are still present, YF476 will be continued, but the dose may be increased to 75 or 100 mg *once* daily, at the discretion of the investigator. If a satisfactory response is seen with 50 mg YF476 *once* daily after 6 weeks for the first few patients, subsequent patients may be started on 25 mg YF476 *once* daily.

Visit 5 will be 9 weeks after starting YF476, to assess tolerability, safety and blood levels of gastrin, CgA and YF476.

Visit 6 will be 12 weeks after starting YF476, to assess tolerability and safety, to repeat the gastroscopy and gastric biopsies, and to take blood samples to assess levels of gastrin, CgA and YF476. If the carcinoids have not regressed, the patient will leave the trial. If the carcinoids have regressed, the patient will proceed to Visit 7.

Visit 7 will be a follow-up visit, 12 weeks after the end of treatment with YF476, only for patients who respond to YF476. The gastroscopy and blood sampling (for gastrin and CgA) will be repeated.

Extended treatment

Some patients will be eligible for extended treatment with YF476, as described below. To be eligible for further treatment, the patient must have benefitted from 12 weeks' treatment, and have tolerated YF476 well, and must give fully informed written consent.

Visit 8 will be to inform the patient about the extended dosing period and prescribe YF476 capsules. If the investigator thinks that a new baseline gastroscopy and blood sampling (for safety, gastrin and CgA) is necessary, this will be carried out.

Visit 9 will be 12 weeks after starting extended treatment with YF476, to assess tolerability, safety and blood levels of gastrin, CgA and YF476.

Visit 10 will be 24 weeks after starting extended treatment with YF476, to assess tolerability and safety, to repeat the gastroscopy and gastric biopsies, and to take blood samples to assess blood levels of gastrin, CgA and YF476.

Visit 11 will be 36 weeks after starting extended treatment with YF476, to assess tolerability, safety and blood levels of gastrin, CgA and YF476.

Visit 12 will be 52 weeks after starting extended treatment with YF476, to assess tolerability and safety, to repeat the gastroscopy and gastric biopsies, and to take blood samples to assess levels of gastrin, CgA and YF476.

A formal follow-up visit will be omitted, as the patients are closely followed by the investigator as part of their standard healthcare.

The end of the trial will be the last visit by the last subject.

2.7 Methods

- Gastroscopy with photographs and video of carcinoids, and biopsies of antrum, corpus and carcinoids;
- Blood samples for assay of gastrin, CgA, and YF476, and serum for storage;
- Diary card: adverse events, concomitant medication and compliance; and
- Tolerability and safety assessments.

2.8 Variables

- Visual assessment of the number, size and distribution of gastric carcinoids;
- Histology and real-time PCR of biopsies: for ECL-cell markers and biomarkers (such as CgA);
- Fasting serum gastrin and plasma CgA concentrations;
- Trough and peak serum YF476 concentrations; and
- Medical examination; ECG; vital signs; adverse events; and safety tests of blood and urine.

3 Contents

1 Signatures 2

2 Summary 3

2.1 Background 3
2.2 Trial medication 3
2.3 Objectives 3
2.4 Type of trial 3
2.5 Trial population 3
2.6 Trial design 4
2.7 Methods 6
2.8 Variables 6

3 Contents 7

4 List of abbreviations 10

5 Trial personnel 12

5.1 Planned sites and principal investigators 13

6 Trial flow charts 14

6.1 Trial overview 14
6.2 Screening and treatment periods 15
6.3 Flowchart for extended treatment 16

7 Introduction 17

7.1 Gastrin 17
7.2 Hypergastrinaemia and its effect on ECL cells 17
7.3 Role of gastrin in cell biology and carcinogenesis 18
7.4 Gastric carcinoids 19
7.4.1 Type I gastric carcinoids 19
7.4.2 Type II gastric carcinoids 20
7.4.3 Type III gastric carcinoids 20
7.5 Proton pump inhibitors and gastric carcinoids 20
7.6 Gastrin receptor antagonists 21
7.7 Effect of a gastrin receptor antagonist on ECL cells and on ECL-cell hyperplasia induced by a proton pump inhibitor 22
7.8 Review of YF476 22
7.8.1 Background 22
7.8.2 Chemistry 23
7.8.3 Non-clinical pharmacology 23
7.8.4 Toxicology and other non-clinical studies 23

7.8.5 Completed studies in healthy subjects 26
7.8.6 Studies in progress in healthy subjects 28
7.9 Rationale for this study, and choice of study design 28

8 Objectives 32

9 Overall trial design 32

10 Trial population 33
10.1 Planned number of subjects 33
10.2 Inclusion criteria 33
10.3 Exclusion criteria 34
10.4 Withdrawal of subjects from the trial 34

11 Treatments 35
11.1 Treatments administered 35
11.2 Labelling 35
11.3 Selection and timing of dose for each subject 35
11.4 Previous and concomitant treatment 35
11.5 Assessment of compliance 36

12 Main outcome measures 36
12.1 Efficacy 36
12.2 Safety and tolerability 36

13 Dietary and lifestyle restrictions 36

14 Procedures and observations 37
14.1 Visit 1: Screening for eligibility (part 1) 37
14.2 Visit 2: Screening for eligibility (part 2), and start of YF476 treatment 37
14.3 Visit 3: 3 weeks after starting YF476 38
14.4 Visit 4: 6 weeks after starting YF476 38
14.5 Visit 5: 9 weeks after starting YF476 39
14.6 Visit 6: 12 weeks after starting YF476 40
14.7 Visit 7: 12 weeks after stopping YF476 40
14.8 Visit 8 40
14.9 Visit 9: 12 weeks after starting extended YF476 dosing 41
14.10 Visit 10: 24 weeks after starting extended YF476 dosing 41
14.11 Visit 11: 36 weeks after starting extended YF476 dosing 42
14.12 Visit 12: 52 weeks after starting extended YF476 dosing 42
14.13 Flexibility of study days 42
14.14 Methods 43
14.15 Total volume of blood removed 45
14.16 Precautions 45

15 Trial materials 45
15.1 Active pharmaceutical ingredient (API): YF476 45
15.2 Formulated intermediate: spray-dried YF476 and HPMC 46
15.3 Final formulation 47
15.3.1 Packaging and labelling 47
15.3.2 Storage and accountability of IMP 47
16 Adverse events 48
16.1 Definitions of adverse events 48
16.2 Procedures for recording adverse events 49
16.3 Procedures for dealing with serious adverse events 50
16.4 Procedures for handling withdrawals due to adverse events 51
17 Data management and quality assurance 51
18 Statistical methods 52
18.1 Statistical analyses 52
18.2 Determination of sample size 53
19 Ethical and regulatory requirements 53
20 Trial documentation 54
20.1 Protocol amendments 54
20.2 Case report forms 56
20.3 Reporting of results 56
21 Obligations of the sponsor and investigator 57
21.1 Monitoring and auditing 57
21.2 Compensation of patients 57
21.3 Confidentiality 57
21.4 Publication 57
21.5 Archiving 58
22 Premature termination of the trial 58
23 References 58
Appendix 1 Restricted medication 67
Cytochrome P450 67
Assessment and management of risk of potential drug-drug interactions 68
Guidance for investigators 69

4 List of abbreviations

ABPI	Association of the British Pharmaceutical Industry
ALT	alanine aminotransferase
AST	aspartate aminotransferase
AUC	area under the curve
BP	blood pressure
°C	degrees Celsius
CAG	chronic atrophic gastritis
CCK-B	cholecystokinin B
CgA	chromogranin A
cm	centimetre
CRF	case report form
CYP	cytochrome P450 enzyme
D	decreased by more than predetermined amount from screening value, or Day, according to context
ECG	electrocardiogram
ECL cell	enterochromaffin-like cell
EDTA	ethylenediamine tetraacetic acid
FDA	Food and Drug Administration
G	gauge or G force, according to context
g	gram
GCP	Good Clinical Practice
GP	General Practitioner
gamma-GT	gamma glutamyl transpeptidase
GMP	Good Manufacturing Practice
GORD	gastro-oesophageal reflux disease
GRA	gastrin receptor antagonist
GT	glutamyl transpeptidase
H	higher than normal range
HDC	histidine decarboxylase
H2RA	histamine H_2-receptor antagonist
h	hour
Hb	haemoglobin
H. pylori	*Helicobacter pylori*
hERG	human ether-à-go-go related gene
HMR	Hammersmith Medicines Research
I	increased by more than predetermined amount from screening value
ICH	International Conference on Harmonisation of Technical Requirements for Registration of Pharmaceuticals for Human Use
IC_{50}	concentration of drug causing 50% inhibition of a response
IMP	investigational medicinal product
ISO	International Standards Organization
kg	kilogram
L	lower than normal range or litre, according to context
m	metre
MEN 1	multiple endocrine neoplasia-1 gene
MIA(IMP)	Manufacturer's Authorisation for Investigational Medicinal Products

MCV	mean corpuscular volume
MedDRA	Medical Dictionary for Regulatory Activities
MHRA	Medicines and Healthcare products Regulatory Agency
mg	milligram
min	minute or minimum, according to context
mL	millilitre
mm	millimetre
mm Hg	millimetres of mercury
MMP-7	matrix metalloproteinase-7
mV	millivolts
ng	nanogram
NOAEL	no-observable-adverse-effect level
NRES	National Research Ethics Service
NSAID	non-steroidal anti-inflammatory drug
PC	personal computer
PCR	polymerase chain reaction
pH	the negative logarithm to base 10 of the hydrogen ion concentration
PST	pancreastatin
PPI	proton pump inhibitor
QA	Quality Assurance
QTc	interval of the ECG corrected for heart rate
REC	Research Ethics Committee
SAS	statistical analysis software
SST	somatostatin
sec	second
SUSAR	suspected unexpected serious adverse reaction
uPA	urokinase-type plasminogen activator
VMAT	vesicular monoamine transporter
WBC	white blood cells
ZES	Zollinger-Ellison syndrome
μg	microgram
μmol	micromole

5 Trial personnel

**Principal investigator**	Professor Mark Pritchard PhD FRCP<br>Professor of Gastroenterology<br>Honorary Consultant Gastroenterologist<br>Gastroenterology Unit<br>Royal Liverpool University Hospital<br>Prescot Street<br>Liverpool L7 8XP<br>Tel: 0151 794 5772 Fax: 0151 794 6825<br>Email: mark.pritchard@liverpool.ac.uk
**Sponsor's medical monitor**	Dr Malcolm Boyce BSc FRCP FFPM<br>Trio Medicines Ltd<br>PO Box 53346<br>London NW10 7XU<br>UK<br>Tel: 020 8961 4130 Fax: 020 8961 8665<br>Email: mboyce@hmrlondon.com
**Clinical Project Manager**	Dr Gillian Turnbull MA (Oxon) PhD<br>Hammersmith Medicines Research (HMR)<br>Cumberland Avenue<br>Park Royal<br>London NW10 7EW
**Statistician**	Mrs Toni Mitchell BSc MSc CStat<br>Head of Statistics and Data Management<br>HMR
**Central laboratory**	Johannes Stanta PhD<br>Head of Laboratory Services<br>HMR Laboratory
**Pharmacokinetic assays**	Professor Atholl Johnston BSc PhD MRCPath<br>Analytical Services International Ltd<br>St George's University of London, Cranmer Terrace<br>London SW17 0RE

5.1 Planned sites and principal investigators

Principal investigator

Professor Mark Pritchard PhD FRCP
Professor of Gastroenterology
Honorary Consultant Gastroenterologist
Gastroenterology Unit
Royal Liverpool University Hospital
Prescot Street
Liverpool L7 8XP
Tel: 0151 794 5772 Fax: 0151 794 6825
Email: mark.pritchard@liverpool.ac.uk

6 Trial flow charts

6.1 Trial overview

Visit 1[1]	Visit 2[1]	Visit 3	Visit 4[2]	Visit 5	Visit 6[3]	Visit 7
Screening (basic)	Screening (gastroscopy) & start 50 mg YF476 once daily	Safety & efficacy check after 3 weeks of YF476	Safety & efficacy check & gastroscopy after 6 weeks of YF476	Safety & efficacy check after 9 weeks of YF476	Safety & efficacy check & gastroscopy after 12 weeks of YF476	Follow up 12 weeks after stopping YF476

1. It might be possible to combine Visits 1 & 2 for some patients.
2. If gastroscopy at Visit 4 reveals complete regression of gastric carcinoids, YF476 will be stopped, Visits 5 & 6 omitted, and the patient will proceed to Visit 7. If gastroscopy at Visit 4 reveals continuing presence of gastric carcinoids, the dose of YF476 can be increased from 50 mg *once* daily to 75 or 100 mg *once* daily.
3. If gastroscopy at Visit 6 reveals that gastric carcinoids have not regressed, Visit 7 will be omitted.

6.2 Screening and treatment periods

	Visit 1: Screening	Visit 2: Screening[3]	Treatment period				Visit 7: 12 weeks after stopping YF476
			Visit 3: 3 weeks	Visit 4: 6 weeks	Visit 5: 9 weeks	Visit 6: 12 weeks	
Informed consent	X						
Medical history	X						
Inform GP	X						
Medical examination	X			X		X	
12-lead ECG	X		X	X	X	X	
Vital signs	X		X	X	X	X	
Safety tests of blood & urine	X		X	X	X	X	
Fasting serum gastrin, plasma CgA and serum for storage	X		X	X	X	X	X
Plasma YF476			X[7]	X[7]	X[7]	X[7]	
Pregnancy test[1]	X	X	X	X	X	X	
H. pylori test[6]	X						
Gastroscopy[2]		X		X[4]		X[5]	X[5]
Collect & check diary card			X	X	X	X	
Collect & check YF476 container for compliance			X	X	X	X	
Issue YF476 capsules		X	X	X[4]	X[4]		
Issue diary card		X	X	X	X		
AE & concomitant medication enquiry			<-----------------------X----------------------->				

1. Women of child-bearing potential
2. Fasting from food and fluids for at least 6 hours before gastroscopy
3. Patients who meet the entry criteria, start 50 mg YF476 by mouth once daily at home on the day after Visit 2.
4. If gastroscopy at Visit 4 reveals complete regression of carcinoids on visual inspection, stop YF476, omit Visits 5 & 6, and proceed to Visit 7. If gastroscopy at Visit 4 reveals continuing presence of carcinoids on visual inspection, increase dose of YF476 to 75 or 100 mg *once* daily (optional).
5. If gastroscopy at Visit 6 reveals no regression of carcinoids, omit Visit 7.
6. *H. pylori* test will be done according to local SOPs, only if status is unknown.
7. Patients fast overnight before visit and bring their container of YF476 with them. They take YF476 only after collection of blood samples for trough YF476, fasting serum gastrin and CgA levels, and gastroscopy (if applicable). Patients stay 1 h after dosing for collection of blood sample for peak YF476 level

6.3 Flowchart for extended treatment

Visit 8[3]	Visit 9[1,3]	Visit 10[1,3]	Visit 11[1,3]	Visit 12[1]
Consent to extended dosing. Some patients[5] might have a new baseline gastroscopy and blood sampling[6].	Safety & efficacy check[2] after **12 weeks** of YF476	Safety & efficacy check[2] & gastroscopy[4] after **24 weeks** of YF476	Safety & efficacy check[2] after **36 weeks** of YF476	Safety & efficacy check[2] & gastroscopy[4] after **52 weeks** of YF476

1. Patients fast overnight before the visit and bring their container of YF476 with them.
2. The investigator will carry out the following procedures: Medical examination, 12-lead ECG, vital signs, safety tests of blood and urine, fasting serum gastrin, plasma CgA and serum for storage, plasma YF476 (before and 1 hour after dosing), and pregnancy test (women of child-bearing potential only). The investigator will collect and check diary card, collect and check YF476 container for compliance, and ask the patient about adverse events and concomitant medication.
3. The investigator will issue YF476 capsules and diary card.
4. Patients must fast from food and fluid for at least 6 hours before gastroscopy. They will take YF476 only after the gastroscopy.
5. If the investigator thinks that a new baseline gastroscopy is needed, the patient will be informed of this before visit 8, and must fast from food and fluid for at least 6 hours before gastroscopy.
6. Blood sampling to include safety tests, fasting serum gastrin and plasma CgA, if the investigator thinks it is necessary.

7 Introduction

7.1 Gastrin

Gastrin is a hormone secreted by G cells in the gastric antrum (Dockray *et al* 2001). It has at least two major physiological functions: to stimulate gastric acid secretion and to stimulate growth of the oxyntic mucosa (Håkanson *et al* 1994).

Food stimulates G cells to secrete gastrin into the circulation (Richardson *et al* 1976; DelValle and Yamada 1990). Large increases in blood levels of gastrin occur after food. Circulating gastrin stimulates gastrin receptors (also called CCK-B or CCK-2 receptors) on the enterochromaffin-like (ECL) cells in the gastric fundus, causing those cells to secrete histamine (Konagaya *et al* 2001; Bakke *et al* 2001), which in turn stimulates adjacent gastric oxyntic cells to secrete acid into the lumen of the stomach. Gastric acid secretion is controlled by the action of (H^+, K^+)–ATPase (the proton pump) in response to stimulation of muscarinic M_3 receptors (Aihara *et al* 2003) or histamine H_2 receptors (Hersey and Sachs 1995).

Initially, acid secretion is neutralised by the buffering capacity of food. But, as digestion proceeds and the gastric contents are moved into the duodenum, the buffering capacity of the food diminishes and intragastric pH starts to fall. Falling pH stimulates antral D cells to secrete somatostatin (SST), a hormone that switches off gastrin secretion by the G cells (Sanduleanu *et al* 2001).

Acid secretion is also under nervous control, by the vagus. Food causes central stimulation of the vagus, which leads to gastrin release from G cells in the stomach and thereby an increase in acid secretion – the cephalic phase of gastric acid secretion.

7.2 Hypergastrinaemia and its effect on ECL cells

Anything that reduces acid secretion – such as atrophic gastritis, a proton pump inhibitor (PPI) or a histamine H_2-receptor antagonist (H2RA) – interferes with the normal switching off of G-cell secretion, and so serum gastrin rises.

The effects of PPI-induced hypergastrinaemia have been studied most in the rat. Gastrin stimulates the release into the circulation of histamine and pancreastatin (PST), a peptide derived from chromogranin A (CgA), and increases the synthesis of CgA and the histamine-forming enzyme histidine decarboxylase (HDC) in ECL cells (Håkanson *et al* 1993). Serum PST (Håkanson *et al* 1995) and CgA (Sanduleanu *et al* 2001; Syversen *et al* 1994) are markers of ECL-cell activity. A study in muscarinic M_3 receptor knock-out mice has shown that the M_3 receptor is essential for the trophic response to hypergastrinaemia (Aihara *et al* 2003).

The time-course of PPI-induced histological changes in rat ECL cells is: hyperactivity (histamine/PST release) within minutes; new HDC synthesis within hours; hypertrophy (increase in endoplasmic reticulum and Golgi size) within days;

hyperplasia in weeks/months; and dysplasia/carcinoids in about 2 years. Hyperplasia occurs mainly after 4 to 10 weeks, but takes longer to develop fully (Håkanson *et al* 1993; Chen *et al* 1996). The gradual transformation of ECL cells secondary to hypergastrinaemia (Qvigstad *et al* 1999) is summarised below.

Hypergastrinaemia

↓

Stimulation of ECL-cell proliferation

↓

Diffuse ECL-cell hyperplasia

↓

Focal / micronodular hyperplasia

↓

Dysplasia

↓

ECL-cell neoplasia

PPI – omeprazole, esomeprazole, lansoprazole, pantoprazole and rabeprazole – are mainly used to treat patients with peptic ulcer disease or gastro-oesophageal reflux disease (GORD). But, after *H. pylori* eradication was introduced, the need for maintenance therapy for peptic ulcer disease was largely eliminated, and GORD became the main indication for prolonged gastric acid inhibition. PPI have been given to many millions of patients since the first PPI, omeprazole, was marketed in the 1980s. Although PPI are remarkably well tolerated, there has always been concern that prolonged hypergastrinaemia might eventually lead to development of ECL-cell carcinoids in patients (Waldum *et al* 2002), as in rats. But, there is no evidence from studies of patients on long-term treatment with PPI that they cause gastric carcinoids (Laine *et al* 2000).

7.3 Role of gastrin in cell biology and carcinogenesis

Gastrin mediates gene expression that is associated with cell division, invasion, angiogenesis and anti-apoptotic activity. All of those are pivotal in increasing malignant potential (Watson *et al* 2006). Also, the source of gastrin can be either endocrine in nature, through gastric mucosal secretion, or of tumour origin, through mutation of the gastrin gene. Gastrin is regulated by a positive regulator, gastrin-releasing peptide, and a negative regulator, somatostatin. Hypergastrinaemia promotes the development of adenocarcinoma in mouse models, whereas in humans – such as patients with atrophic gastritis or Zollinger-Ellison syndrome (ZES) – hypergastrinaemia results in ECL-cell hyperplasia and gastric carcinoids.

7.4 Gastric carcinoids

Carcinoids are a subset of tumours with features of neuroendocrine differentiation. Most arise within the gastrointestinal tract. Gastric tumours of neuroendocrine origin are derived from, or differentiate towards, the ECL cells of the gastric corpus. 562 (4.1%) of a total of 13,715 recorded carcinoids were gastric in origin (Modlin *et al* 2003 and 2004). Rindi *et al* (1993) separated gastric carcinoids into three types. The table below lists their main characteristics (Burkitt and Pritchard 2006).

**Characteristic**	**Type I**	**Type II**	**Type III**
**Associated disease**	Chronic atrophic gastritis and pernicious anaemia	Zollinger-Ellison syndrome and MEN type I	None
**Proportion of tumours**	80%	5%	15%
**Site of tumours**	Fundus	Fundus (sometimes antrum)	Antrum or fundus
**Number of tumours**	Multiple	Multiple	Single
**Size of tumour**	<1 cm	<1cm	2–5cm
**Plasma gastrin level**	High	High	Normal
**Gastric acid output**	Low	High	Normal
**Prognosis**	Good	Mostly good; 30% metastasise	Poor; >50% metastasise

7.4.1 Type I gastric carcinoids

About 5% of patients with chronic atrophic gastritis and hypergastrinaemia develop type I gastric carcinoids. About 80% of all gastric carcinoids are type I. Atrophic gastritis is characterised by chronic inflammation of the gastric mucosa with loss of gastric glandular cells and replacement by intestinal-type epithelium, pyloric-type glands and fibrous tissue. It can result in oxyntic mucosal atrophy, loss of parietal and chief cells, and achlorhydria. Achlorhydria stimulates G-cell hyperplasia, which results in hypergastrinaemia. Hypergastrinaemia causes ECL-cell hyperplasia which may lead to growth of gastric carcinoids (Dakin *et al* 2006). Atrophic gastritis is associated with serum antibodies to parietal cells and intrinsic factor, and is regarded as an autoimmune disease. Intrinsic factor deficiency causes vitamin B-12 malabsorption. Eventually, some patients develop pernicious anaemia with haematological, gastrointestinal and neurological complications. Patients with pernicious anaemia also have a 3-fold increase in gastric adenocarcinoma (Sepulveda 2006; Borch 1989).

Most publications report that type I carcinoids have a good prognosis (Borch *et al*

2005; Rindi 1996; Rappel *et al* 1995). However, even type I tumours have the potential to metastasise (Borch 1989; Ahlman *et al* 1994; Kaltsas *et al* 2004; Qvigstad *et al* 1999). If a carcinoid spreads locally or metastasises, then it may well prove life-threatening and require surgery.

7.4.2 Type II gastric carcinoids

Type II carcinoids occur in patients with Zollinger-Ellison syndrome (ZES). ZES is a condition in which one or more gastrinomas – usually in the small intestine or pancreas – secrete excessive gastrin, resulting in hypergastrinaemia (Ellison *et al* 2006). Hypergastrinaemia causes hyperplasia and hypersecretion of the acid-secreting cells of the stomach and severe peptic ulcer disease. Hypergastrinaemia also causes ECL-cell hyperplasia, which can lead to growth of type II carcinoids (Pellicano *et al* 2006).

Of 106 patients with gastrinoma (Ellison *et al* 2006), 80 had sporadic gastrinoma and 26 had Multiple Endocrine Neoplasia-1 gene (MEN-1) mutation. Patients with ZES and MEN-1 have a 20–30 fold higher chance of developing a gastric carcinoid than patients with sporadic ZES (Jensen and Fraker 1994). Up to 20% of patients with ZES and MEN-1 develop type II gastric carcinoids (Burkitt and Pritchard 2006). MEN-1 is an autosomal dominant mutation in the MEN-1 gene, which codes for the tumour suppressing protein, MENIN. Although hypergastrinaemia is the primary initiator for type II carcinoids, cofactors such as MEN-1 are also required for their development (Richards *et al* 2004). Loss of heterozygosity for MEN-1 is also found in many type I and type II carcinoids (D'Adda *et al* 1999).

Patients with ZES suffer mainly from the effects of severe ulcer disease – dyspepsia, perforation, bleeding, diarrhoea and weight loss (Roy *et al* 2000) – caused by hypergastrinaemia-induced hypersecretion of gastric acid (Norton *et al* 1992).

The trophic effects of prolonged hypergastrinaemia on ECL cells lead to formation of gastric carcinoids (Borch *et al* 2005). About 30% of type II carcinoids metastasise (Burkitt and Pritchard 2006). Gastrinomas and gastric carcinoids both have the potential to metastasise.

7.4.3 Type III gastric carcinoids

Type III gastric carcinoids are sometimes referred to as sporadic gastric carcinoids. They are usually large single tumours and their potential for malignancy is high (Burkitt and Pritchard 2006). They are not associated with hypergastrinaemia.

7.5 Proton pump inhibitors and gastric carcinoids

Most patients taking a PPI develop hypergastrinaemia (Jansen *et al* 1990; Lamberts *et al* 1993). Serum gastrin levels can be as high as those in Zollinger-Ellison syndrome

(Ligumsky *et al* 2001). The increase in the use of PPI since the late 1980s has coincided with a six-fold increase in the incidence of gastric carcinoids in the USA (Modlin *et al* 2004; Hodgson *et al* 2005) and England (unpublished data from National Cancer Registry for England). Moreover, the incidence of all gastric cancers has actually decreased over this period. This apparent increase in gastric carcinoids may be an artefact due to improved detection and diagnostic tests. However, a comparison with the incidence of the gastric hyperplastic polyp suggests otherwise (Burkitt and Pritchard 2006). A three-fold increase in the incidence of gastric hyperplastic polyp cases occurred between 1982 and 2002 (Laxen *et al* 1982; Ljubicic *et al* 2002). That is half of the increase in the incidence of gastric carcinoids, suggesting that the widespread use of PPI might be contributing to the rising incidence of type II carcinoids. Most ZES patients are treated with a PPI to control their hyperacidity, even if surgery to remove a gastrinoma is successful. The PPI may further increase circulating gastrin and encourage the formation of type II carcinoids. But, the evidence that long-term use of PPI in patients with gastrinomas contributes to the development of gastric carcinoids is inconclusive (Jensen 2006). However, PPI-induced hypergastrinaemia leads to rebound hyperacidity on stopping the PPI (Nishida *et al* 1995; Waldum *et al* 1996; Gillen *et al* 1999; Gillen and McColl 2001; Qvigstad *et al* 2004; Fossmark *et al* 2005;Reimer *et al* 2009; Niklasson *et al* 2008), so the hypergastrinaemia cannot be entirely harmless.

7.6 Gastrin receptor antagonists

Many gastrin receptor antagonists (GRA) have been described (Håkanson *et al* 1999; McDonald 2001; Black and Kalindjian 2002) but none marketed. Most have had drawbacks, mainly: low affinity for the gastrin receptor; poor selectivity for the gastrin receptor; limited bioavailability; and inactivity when given by mouth. In contrast, YF476 is a potent, orally active, highly selective and competitive antagonist of gastrin receptors (Semple *et al* 1997). It blocks the effects of gastrin *in vitro* (IC_{50} 10.0 nM) and *in vivo* (Takinami *et al* 1997; Takemoto *et al* 1998), and is regarded as the 'gold standard' among GRA (Black and Kalindjian 2002; Ding *et al* 1997). Like PPI, YF476 produces sustained and almost complete inhibition of gastric acid secretion in rats.

A PubMed search currently yields 27 non-clinical papers on YF476. Several of them show that YF476 prevents the carcinogenic effects of gastrin in animal models (Martinsen *et al* 2003; Takaishi *et al* 2005; Zhao *et al* 2005; Cui *et al* 2006).

Three GRA have been studied in healthy subjects: L-365,260 (Murphy *et al* 1993); CR2194 (Beltinger *et al* 1999); and YF476 (Boyce *et al* 2000a, 2000b, 2002 and 2004). Gastrazole has been given to patients with pancreatic cancer for up to 1 year (Chau *et al* 2006; Black 2009). But, no GRA has been marketed.

7.7 Effect of a gastrin receptor antagonist on ECL cells and on ECL-cell hyperplasia induced by a proton pump inhibitor

Eissele *et al* (1992) first showed that a GRA prevented ECL-cell hyperplasia induced by PPI. Ding *et al* (1997) assessed the effects in rats of short intravenous infusions of three GRA, including YF476, on ECL cells stimulated by intravenous gastrin or the hypergastrinaemia induced by omeprazole. YF476, which was the most potent compound, caused dose-dependent antagonism of gastrin-evoked HDC activation. Also, it antagonised omeprazole-induced HDC activation and the gastrin- and omeprazole-induced rise in serum PST.

Chen *et al* (2000) gave rats YF476 for up to 8 weeks to assess their effects on normal ECL cells and on ECL cells exposed to hypergastrinaemia induced by omeprazole. Changes in ECL-cell morphology were assessed by immuno-cytochemistry and electron microscopy, and changes in ECL cell-related biochemical variables were monitored by measuring serum PST and oxyntic mucosal PST and HDC activity.

YF476 had little if any effect on the density of normal ECL cells, but transformed them from slender, elongated cells with prominent projections to small, spherical cells without projections. The Golgi complex, rough endoplasmic reticulum and secretory granules were reduced in size. Serum pancreastatin and oxyntic mucosal HDC activity were lowered within hours.

YF476 prevented the effects of omeprazole-induced hypergastrinaemia on ECL-cell activity and density. Omeprazole increased the thickness of the oxyntic mucosa whereas YF476 reduced the thickness of the oxyntic mucosa, similar to that seen in fasting animals, and prevented the increase in thickness induced by omeprazole.

We have done a study of YF476 and rabeprazole, alone and in combination for 6 weeks in healthy subjects, using plasma CgA as a biomarker for ECL-cell activity. Rabeprazole by itself caused a large increase in plasma CgA whereas YF476 by itself did not affect plasma CgA. When combined with rabeprazole, YF476 abolished the plasma CgA response to rabeprazole by itself (See YF476 Investigator's Brochure, 2009).

7.8 Review of YF476

7.8.1 Background

YF476 was discovered in the UK by Ferring, a Danish pharmaceutical company. Ferring has licensed YF476 to Trio Medicines Ltd for clinical development. TRIO is the sponsor of the study. TRIO has delegated its roles and responsibilities to Hammersmith Medicines Research Ltd (HMR), a contract research organisation.

7.8.2 Chemistry

YF476 was developed from a series of benzodiazepine compounds (Semple e*t al* 1997). The chemical formula is (R)-1-[2,3-dihydro-2-oxo-1-pivaloylmethyl-5-(2-pyridyl)-1 *H* -1,4- benzo-diazepin-3yl]-3-(3-methylaminophenyl)urea.

7.8.3 Non-clinical pharmacology

Studies *in vitro* and *in vivo* have shown YF476 to be a potent, highly selective, and competitive antagonist of gastrin receptors (Takinami *et al* 1997; Takemoto *et al* 1998).

YF476 inhibited specific binding to cloned human gastrin receptors *in vitro*. In rats, intravenous YF476 caused dose-dependent inhibition of pentagastrin-stimulated gastric acid secretion but did not affect histamine- or bethanochol-induced secretion at high doses. In dogs with a chronic gastric fistula, intravenous and oral YF476 caused dose-dependent inhibition of pentagastrin-stimulated gastric acid output and was more potent than famotidine, an H2RA. Oral YF476 also caused dose-dependent inhibition of peptone-stimulated gastric acid secretion in dogs with a chronic fistula, and was more potent than either famotidine or omeprazole.

Inhibition of basal and pentagastrin-stimulated gastric acid by YF476 on the first day of dosing persisted after repeated daily doses in rats with a gastric fistula (YF476 Investigator's brochure, 2009). The effects of a large, single dose of subcutaneous YF476 were also long lasting (Kitano *et al* 2000).

7.8.4 Toxicology and other non-clinical studies

Toxicology studies of YF476 consisted of: single- and/or repeat-dose toxicity studies in rats and dogs; toxicokinetics in rats and dogs; and embryo-foetal toxicity studies in rats and rabbits. Other non-clinical studies consisted of: safety pharmacology studies in rats, mice and dogs; genotoxicity studies *in vivo* and *in vitro*; studies of metabolism by cytochrome P450 in human liver microsomes *in vitro*; a study to determine the extent of YF476 protein binding in normal human serum *in vitro*; a study of the effect of YF476 on the hERG ion channel in human embryonic kidney cells *in vitro*: and a study to compare the metabolism of ^{14}C-YF476 in rat, dog and human cells *in vitro*.

Toxicology studies

In acute toxicity studies in dogs, 100 and 500 mg/kg/day resulted in similar plasma concentrations of YF476, and caused no adverse effects. Therefore, 100 mg/kg/day was the highest dose tested in a 13-week toxicity study. Changes in pancreas weight and histopathology were observed in some animals, and serum amylase was elevated in one low-dose male. However, there was no clear relationship to dose, and no relationship between histological changes and individual organ weights. So, those changes were considered to be incidental findings, and unlikely to be related to

treatment with YF476. Therefore, 100 mg/kg/day was taken to be the nominal no-observable-adverse-effect level (NOAEL) in dogs.

In a 13-week toxicity study in rats, the NOAEL was 100 mg/kg/day. At 300 and 1000 mg/kg/day, some animals had minor, reversible changes in enzyme activities and heart weight, and discoloured urine and pale faeces. Minor histopathological lesions in the heart and pancreas of 4 treated animals and 1 control animal were observed. Those lesions are part of the background pathology commonly seen in laboratory rodents, so were considered to be spontaneous lesions unrelated to treatment with YF476.

ECG studies

In the 13-week study in dogs, YF476 had no effect on the ECG, and no effect on the ECG intervals, including QTc. *In vitro*, a concentration of YF476 about 4 times the highest C_{max} in a human subject to date did not affect the hERG channel. As about 84% of YF476 is bound to serum protein *in vivo*, that represents 25-fold safety cover.

Cytochrome P450 enzymes

In a study to assess the potential of YF476 to inhibit cytochrome P450 enzymes CYP1A2, CYP2B6, CYP2C19, CYP2D6, CYP2C8, CYP2C9 and CYP3A4 in human liver microsomes *in vitro,* YF476 at a single concentration of ~5 μM inhibited CYP3A4, and to lesser extent CYP2C8 and CYP2C9.

That study did not distinguish between reversible and irreversible inhibition, so a more detailed study was done to do that. In tests for reversible inhibition, the IC_{50} of YF476 was 3.52, 8.37 and 12.1 μM for CYP2C8, CYP3A4 (midazolam) and CYP3A4 (testosterone), respectively. YF476 did not inhibit CYP2C9 at doses up to 25 μM. In tests for irreversible inhibition, 0.5 μM YF476 did not cause significant inhibition of CYP3A4 (midazolam). However, 5 μM YF476 caused 43.9% irreversible inhibition of CYP3A4 (midazolam), which was reduced to 30.2% when corrected for vehicle control. Neither CYP3A4 (testosterone) nor CYP2C9 was irreversibly inhibited by 5 μM YF476.

To assess the likelihood of CYP2C8 and CYP3A4 reversible inhibition in patients *in vivo,* $[I]/K_i$ ratios (where $K_i = IC_{50}/2$, and $I = C_{max}$ for YF476) were calculated from IC_{50} values for reversible inhibition by YF476, and C_{max} for total (bound and unbound) plasma concentrations of YF476 from a study of single doses of 50, 100, 200 and 400 mg in healthy subjects (T–011; see Section 7.8.5). FDA guidelines (2006) state that an interaction is *likely* if the ratio is greater than 1, *possible* if between 1 and 0.1, and *remote* if less than 0.1. Therefore, the likelihood of a drug-drug interaction in patients *in vivo* is:

- CYP2C8: *possible* for 50–400 mg YF476
- CYP3A4 (testosterone): *remote* for 50 & 100 mg YF476, and *possible* for 200 & 400 mg
- CYP3A4 (midazolam): *remote* for 50 mg YF476, and *possible* for 100–400 mg.

YF476 caused some irreversible inhibition of CYP3A4, but the degree of inhibition makes a drug-drug interaction in patients *in vivo* unlikely.

Embryo-foetal studies

Embryo-foetal studies were done in the rat and rabbit, using doses of 100, 300, and 1000 mg/kg/day. YF476 had no effect on embryo-foetal survival, growth or development.

Metabolism

The metabolism of parent ^{14}C-YF476 was most efficiently catalysed by human > dog > rat *in vitro*. Phase I and II metabolites were formed. There was no evidence for formation of unique major human metabolites, although the main metabolite was different from that formed by rat and dog. Thus, the rat and dog are appropriate species to assess the potential toxicity of YF476 in humans.

Further details of non-clinical studies of YF476 are in the Investigator's Brochure.

7.8.5 Completed studies in healthy subjects

HMR has completed and reported 9 studies of YF476 in 162 healthy men and women.

Study code	Design	n	YF476 dose (mg)	Comparator
96-001	single, dose-rising, double-blind, incomplete crossover	15 (M)	0.5–100	placebo
96-016	single-dose, double-blind, 5-way crossover	21 (M/F)	5, 25, 100	placebo & ranitidine 150 mg
96-018	7-day dosing, double-blind, 4 parallel groups	49 (M/F)	25 & 100 bd	omeprazole 20 mg od
97-010	14-day dosing, double-blind, 4 parallel groups	49 (M)	5, 10 & 25 od	placebo
01-022	single-dose, double-blind, 5-way crossover	12 (M/F)	1, 5, 25 & 100	placebo
01-023	7-day dosing, open	10 (M/F)	100 bd	placebo Days 0 & 14
T-001 (05-021)	YF476 & rabeprazole, alone & in combination, 6-weeks' dosing, double-blind, 3 parallel groups	32 (M/F)	100 od	rabeprazole
T-009 (07-013)	single-dose, open, fed-fasted comparison	12 (M/F)	100	–
T-011 (07-024)	new formulation, single, dose-rising, double-blind, fed-fast comparison	10 (M/F)	50–400	placebo

In all studies, YF476 was well tolerated. Adverse events were minor and transient; they resolved spontaneously, and occurred in subjects given placebo as well as those given YF476. There were no effects on vital signs, ECG, or safety tests of blood and urine, and no clinically significant adverse events.

Single doses of YF476 caused dose-dependent increases in 24-h gastric pH. The effect of a single dose of YF476 lasted much longer than that of ranitidine, (an H_2 receptor antagonist). However, the effect of YF476 on 24-h gastric pH was mostly lost after repeated doses, whereas that of omeprazole persisted.

Single doses of YF476 also caused dose-dependent inhibition of pentagastrin-induced falls in gastric pH and increases in gastric volume and H^+ output. Again, the effect on gastric pH was mostly lost after repeated doses of YF476. But, the effects on gastric volume and H^+ output persisted. So, gastrin receptors must be blocked after repeated doses of YF476. YF476 also caused dose-dependent increases in blood gastrin, which persisted after repeated doses, further evidence that gastrin receptors are blocked after repeated doses of YF476.

We do not know the mechanism for the reduced effect of YF476 on gastric pH after repeated doses of YF476, but an effect on buffering of gastric juices seems a possibility. We are doing further studies to assess the mechanism.

There was no evidence of an effect of YF476 on QT interval. However, until we have more experience of YF476, patients with prolonged QT interval should be excluded from clinical trials of YF476 (Shah 2002).

YF476 was measured by the method of Redrup *et al* (2002). In early studies (before 2001), YF476 had linear pharmacokinetics, and consistent bioavailability. Absorption was rapid, and the terminal elimination half-life was about 6 h. AUC_{0-24} after 100 mg, the largest dose studied, was about 650 ng.h/mL, which is about a 5 to 60-fold safety margin in terms of AUC_{0-24} at NOAEL in the 13-week toxicology studies. Steady state was reached by Day 3, and there was no accumulation by Day 7. Only 1–2% was excreted unchanged in the urine.

However, in two subsequent studies (T-001 and T-009), for which we used a different formulation, plasma concentrations of YF476 were only about 10–15% of those achieved in the early studies. Despite the low bioavailability, YF476 showed good pharmacodynamic effects in study T-001 (effects of rabeprazole 40 mg and YF476 100 mg, alone and in combination for 6 weeks, on gastric function). In that study, gastric function was assessed by: 24-h ambulatory gastric pH; pentagastrin-stimulated gastric pH, volume and H+ output; 24-h serum gastrin profile; and 24-h plasma CgA profile (CgA is a biomarker for ECL-cell activity).

On Day 1 or 2, YF476 and rabeprazole by themselves gave similar results. They both:

- inhibited pentagastrin-induced increase in volume of gastric aspirate and H+ secretion;
- increased 24-h ambulatory gastric pH; and
- increased serum gastrin (a biomarker for gastric acid production), but did not affect plasma CgA (a biomarker for ECL-cell activity).

At 6 weeks, YF476:

- was as effective as rabeprazole in reducing gastric acid volume and H+ secretion;
- was as effective as rabeprazole in increasing serum gastrin, a biomarker for gastric acid production;
- did not increase plasma CgA, whereas rabeprazole increased it substantially;
- abolished the rabeprazole -induced increase in CgA; and
- augmented gastric acid suppression by rabeprazole without affecting the increase in gastric pH by rabeprazole.

The combination was more effective than either rabeprazole or YF476 alone in suppressing gastric acid production. Furthermore, the combination did not lose its effect on gastric pH after 6 weeks' dosing. On the contrary, the effect increased.

That YF476 completely suppressed the rabeprazole -induced increase in plasma CgA suggests that YF476 prevented rabeprazole -induced hypergastrinaemia from stimulating the ECL cells. Overall, the effects of YF476 are consistent with antagonism of gastrin receptors.

We conclude that YF476 augments PPI-induced gastric acid suppression and prevents increased ECL-cell activity caused by PPI-induced hypergastrinaemia. Also, whatever the cause of the loss of effect of YF476 on gastric pH after repeated dosing, it is unlikely to impair the ability of YF476 to prevent the unwanted effects of a PPI.

We now have a new formulation of YF476, which was made by spray-drying a solution of YF476 with hydroxypropylmethylcellulose (HPMC). That process generated amorphous YF476 stabilised by an HPMC polymer matrix. We tested single doses (range 50–400 mg) in study T-011. Plasma concentrations were similar to those in the early studies of YF476. After 100 mg of the new formulation, mean C_{max} was 265.3 ng/mL and mean AUC_{0-24} was 527 ng.h/mL. After 400 mg, mean C_{max} was 704.3 ng/mL and mean AUC_{0-24} was 1557 ng.h/mL. The new formulation will be used in this study.

For further information on the non-clinical and clinical studies of YF476, refer to the Investigator's Brochure.

7.8.6 Studies in progress in healthy subjects

Study T-012 (05-029): The aim is to assess the absolute bioavailability of the latest formulation of YF476. 6 subjects received a single oral 100mg dose of YF476 close together with a single intravenous 15 µg dose of ^{14}C–YF476. Blood samples are being assayed for 'cold' YF476 and ^{14}C–YF476. Preliminary results show that the absolute bioavailability of YF476 is about 15%.

Study T-013 (09-013): This study aims to assess the effect of different 14-day dose regimens of YF476 on the increase in circulating CgA induced by a PPI (esomeprazole 40 mg daily for 4 weeks).

To date, 24 subjects have completed the study. Preliminary results show that 25 mg YF476 daily completely suppressed the increase in CgA induced by esomeprazole. YF476 was well tolerated.

7.9 Rationale for this study, and choice of study design

It is clear from the aforementioned information that there are compelling reasons for treating patients with type I or type II gastric carcinoids – both of which are gastrin dependent – with YF476, a gastrin receptor antagonist. Indeed, the European Commission and the USA Food and Drug Administration have designated YF476 an

Orphan Medicinal Product for treatment of patients with gastric carcinoids (European Commission 2007, Food and Drug Administration 2009).

We need to ask ourselves several questions about the trial design.

First, what type of patient should we study?

In this first trial of YF476 in patients with gastric carcinoids, we will study only type I patients for the following reasons. First, the characteristics of patients with type I gastric carcinoids are very different from those of patients with type II gastric carcinoids. Therefore, types I and II gastric carcinoids merit separate trials to assess the efficacy of YF476. Second, there is already some evidence from one patient with type I gastric carcinoids that YF476 will be effective; large multiple gastric carcinoids in a patient with chronic atrophic gastritis, pernicious anaemia and hypergastrinaemia regressed after 4-weeks' intravenous infusion of JB95008, a gastrin receptor antagonist with poor oral bioavailability (Chau *et al* 2006). Several years later, the tumours have not regrown (Thompson, personal communication). Third, although gastric carcinoids are rare tumours, type I are more common and should be less difficult to find. Fourth, the existing treatment of type II patients is likely to be more complicated than that of type I patients. Type II patients will almost certainly be receiving several treatments, such as high-dose PPI and perhaps other therapy to suppress gastric acid secretion – for treatment of peptic ulcer disease – whereas type I patients are likely to be receiving injections of vitamin B12 only – for treatment of pernicious anaemia. Finally, a common protocol for types I and II would be more complicated and not ideal for a first trial of YF476.

Second, what should be the dose regimen of YF476?

Patients with type I gastric carcinoids have raised serum CgA (Borch *et al* 1997; Peracchi *et al* 2005), which is reduced by treatment with octreotide, a somatostatin analogue (Fykse *et al* 2004 and 2005; Campana *et al* 2008; Pregun *et al* 2009). Thus, serum CgA seems to be a useful biomarker for assessing the dose regimen of YF476 for the proposed study. In that respect, we have shown that concomitant administration of 100 mg YF476 daily completely suppressed the huge increase in plasma CgA induced by rabeprazole 20 mg daily for 6 weeks in healthy subjects. We now know that the formulation of YF476 used in that study was poorly bioavailable – 100 mg is equivalent to about 15 mg of the current formulation, so that might be a suitable dose of YF476 for the proposed study. However, patients with chronic atrophic gastritis (CAG) have hypergastrinaemia for many years before they develop gastric carcinoids, so a larger dose of YF476 seems justified. Also, patients with CAG are precious and we don’t want to underdose them. Therefore, we plan to treat patients with 50 mg once daily for up to 12 weeks, and allow the principal investigator to increase the dose after 6 weeks to 75 or 100 mg daily, if necessary. Similar or higher doses of YF476 have been well tolerated in all the completed studies in healthy subjects, so safety should not be rate limiting. Toxicology and toxicokinetic studies support dosing of YF476 for up to 13 weeks. If the response of the one patient with

type 1 gastric carcinoids who was treated with JB95008 (‘Gastrazole’) is typical, treatment with YF476 for less than 12 weeks might be enough.

Food increases the bioavailability of YF476 in healthy subjects, so patients will take YF476 with breakfast.

Third, what tests should we use to assess efficacy?

If YF476 has a marked effect on gastric carcinoids, visual inspection of the tumours at gastroscopy might be enough to assess efficacy in some patients, but we will also take biopsies for histological and biomarker assessments, blood samples to assay fasting serum gastrin and plasma CgA, and trough and peak serumYF476.

Fourth, what should be the study design?

A placebo-controlled trial of a new type of therapy for gastric carcinoids such as YF476 would be the ideal study design. However, the proposed outcome measures are robust enough to assess efficacy in an open trial without a comparator.

Fifth, should we study women as well as men?

Atrophic gastritis is more common in women than men. YF476 is not teratogenic. So, post-menopausal women or pre-menopausal women using a reliable method of contraception (but not a steroid, which is metabolised via CYP3A4; see Appendix 1) will be eligible.

Sixth, should we exclude H. pylori-positive subjects?

Excluding *H. pylori* positive patients would restrict recruitment. We know of no evidence that *H. pylori* infection influences development of gastric carcinoids in humans, so we will test patients for *H. pylori* at screening but not exclude positive subjects. Also, we will assess the impact of infection on the outcome of YF476 treatment.

Seventh, what are the potential risks of harm to the trial subjects from YF476 and from the trial procedures, and how might they be mitigated?

i) Prolongation of QTc interval of the ECG?

YF476 appears to have low potential for prolonging QT interval, a risk factor for torsade de pointes (see Section 7.8.4). But, until we have more information about YF476, patients with prolonged QT interval should be excluded from clinical trials.

ii) Drug-drug interaction?

There is only a remote likelihood of an interaction in patients between YF476 and a drug metabolised through CYP1A2, CYP2B6, CYP2C19, CYP2D6 and CYP2C9 (see Section 7.8.4).

The FDA recommends that, if the [I]/Ki ratio for a CYP enzyme is >0.1 at the highest proposed clinical dose, an interaction study in healthy subjects should be done, or concomitant medication should be restricted appropriately. Currently, the highest

proposed clinical dose of YF476 is 50–100 mg once daily. [I]/Ki ratios are >0.1 for CYP2C8 at YF476 doses of >50 mg and for CYP3A4 at YF476 doses of >100 mg. So, in this study, restriction is required of concomitant medication metabolised by CYP2C8 and possibly also by CYP3A4.

Few drugs are metabolised via CYP2C8, and they are unlikely to be co-administered with YF476. So, it is better to restrict the use of such drugs in early studies of YF476 in patients, rather than carrying out a study of CYP2C8 in vivo.

About 60% of drugs are metabolised via CYP3A4, so that enzyme is more important than CYP2C8. If the therapeutic dose of YF476 proves to be >50–100 mg daily, a study of CYP3A4 in vivo will be done. That will be a study to assess the effect of YF476 on the pharmacokinetics of oral midazolam – a CYP3A4 substrate – in healthy subjects. Meanwhile, only drugs that are metabolised via CYP3A4, and have a wide therapeutic window, may be co-administered with YF476 at the proposed doses in this study.

For guidance, medicines that inhibit or induce CYP3A4/5 and CYP2C8 are listed in Appendix 1 .

Concomitant treatment will be allowed during the proposed trial only if necessary (see Appendix 1). Healthy subjects who were sedated with *intravenous* midazolam before gastroscopy in a recent study (HMR code: 05-021) tolerated it as well after 6 weeks' treatment with YF476 as they tolerated it before baseline gastroscopy (see Section 7.8.5). Therefore, should they wish, patients can be sedated with intravenous midazolam safely before gastroscopy in the proposed trial.

iii) Endoscopy?

Patients who take part in the proposed trial must undergo up to four gastroscopies. All patients will have had at least one gastroscopy before the trial. Most gastroscopies are done without any problem. Some people have a sore throat for a day or so afterwards. Patients may feel tired or drowsy for several hours after midazolam. There is a slightly increased risk of developing a chest infection after gastroscopy. Occasionally, the endoscope damages the gut and causes bleeding, infection, and rarely, perforation.

Finally, what are the potential benefits to patients compared with the risk of harm?

Troublesome gastric carcinoids are usually dealt with surgically (Dakin *et al* 2006). Surgery does not always work and carries a risk of harm. Octreotide – a somatostatin analogue –has also been used to treat patients with type I carcinoids (Fykse *et al* 2004 and 2005). Octreotide has to be given by injection and is not that effective, probably because it is not selective for the gastrin receptor. YF476 is active by mouth and seems to be better tolerated than somatostatin analogues. Therefore, YF476 offers significant benefit over existing treatments for gastric carcinoids.

Overall, the potential benefits to the trial subjects of taking part in the trial greatly outweigh any risks of harm.

8 Objectives

The primary objective of this pilot study is:

- to assess if YF476 is an effective medical treatment for type I gastric carcinoids.

The secondary objectives are:

- to assess the tolerability and safety of YF476; and
- to assess the effect of YF476 on plasma concentration and transcript profiles of biomarkers such as chromogranin A (CgA).

9 Overall trial design

Patients will attend the clinic up to 12 times. The time that patients take to complete the trial will depend on their response to YF476.

At **Visit 1**, patients will be screened (excluding gastroscopy) for eligibility.

At **Visit 2**, patients will undergo gastroscopy and gastric biopsies. If they meet all the inclusion/exclusion criteria, they will start treatment with 50 mg YF476 by mouth *once* daily at home on the next day. Each subject will be given a unique number at the start of treatment.

They will complete a diary card daily throughout the treatment period.

Visit 3 will be 3 weeks after starting YF476, to assess tolerability, safety and blood levels of gastrin, CgA and YF476.

Visit 4 will be 6 weeks after starting YF476, to assess tolerability and safety, to repeat the gastroscopy and gastric biopsies, and to take blood samples to assess levels of gastrin, CgA and YF476. If the gastric carcinoids have regressed completely on visual inspection, YF476 will be stopped, Visits 5 and 6 will be omitted, and the patient will proceed to Visit 7. If the gastric carcinoids are still present, YF476 will be continued, but the dose may be increased to 75 or 100 mg *once* daily, at the discretion of the investigator.

Visit 5 will be 9 weeks after starting YF476, to assess tolerability, safety and blood levels of gastrin, CgA and YF476.

Visit 6 will be 12 weeks after starting YF476, to assess tolerability and safety, to repeat the gastroscopy and gastric biopsies, and to take blood samples to assess levels of gastrin, CgA and YF476. If the carcinoids have not regressed, the patient will leave the trial. If the carcinoids have regressed, the patients will proceed to Visit 7.

Visit 7 will be a follow-up visit, 12 weeks after the end of treatment with YF476, only for patients who respond to YF476. The gastroscopy and blood sampling for fasting gastrin and CgA will be repeated.

Extended treatment

Some patients will be eligible for extended treatment with YF476, as described below. To be eligible for further treatment, the patient must have benefitted from 12 weeks' treatment, and have tolerated YF476 well, and must give fully informed written consent.

Visit 8 will be to inform the patient about the extended dosing period and prescribe YF476 capsules. If the investigator thinks that a new baseline gastroscopy and blood sampling (for safety, gastrin and CgA) is necessary, this will be carried out.

Visit 9 will be 12 weeks after starting extended treatment with YF476, to assess tolerability, safety and blood levels of gastrin, CgA and YF476.

Visit 10 will be 24 weeks after starting extended treatment with YF476, to assess tolerability and safety, to repeat the gastroscopy and gastric biopsies, and to take blood samples to assess blood levels of gastrin, CgA and YF476.

Visit 11 will be 36 weeks after starting extended treatment with YF476, to assess tolerability, safety and blood levels of gastrin, CgA and YF476.

Visit 12 will be 52 weeks after starting extended treatment with YF476, to assess tolerability and safety, to repeat the gastroscopy and gastric biopsies, and to take blood samples to assess levels of gastrin, CgA and YF476.

A formal follow-up visit will be omitted, as the patients are closely followed by the investigator as part of their standard healthcare.

The end of the trial will be the last visit by the last subject.

10 Trial population

10.1 Planned number of subjects

Up to 10 eligible men or women will complete the trial.

10.2 Inclusion criteria

1. Patients known to have gastric carcinoids associated with chronic atrophic gastritis and hypergastrinaemia and who attend the out-patient clinic of the principal investigator.
2. Men; post-menopausal women; pre-menopausal women who have been sterilised by tubal ligation, hysterectomy or bilateral oophorectomy; or pre-menopausal

women using one of the allowed methods of contraception: condom and spermicide or intra-uterine device.

3. Adults ≥18 years.
4. Good general health.
5. Able to give fully-informed, written consent.

10.3 Exclusion criteria

1. Women who are pregnant, lactating or using a steroid contraceptive.
2. History of gastric surgery, apart from surgery for gastric carcinoids.
3. Evidence of Zollinger-Ellison syndrome.
4. Prolonged QTc interval (> 450 msec).
5. Certain medicines and herbal remedies (see Appendix 1) taken during the 7 days before Visit 1.
6. Previous treatment with somatostatin analogues.
7. Participation in a clinical trial of an unlicensed medicine within the previous 3 months.

10.4 Withdrawal of subjects from the trial

Subjects are free to withdraw from the trial at any time without giving reasons. Furthermore, the investigator may withdraw a subject for medical reasons such as intolerance to trial medication, intercurrent illness, need for medication which is contraindicated, lack of efficacy of trial medication, or withdrawal of consent. The investigator will assess reasons for withdrawal as far as possible and will fully record the circumstances and medical details.

Subjects will be informed before they agree to take part in the trial that, if they withdraw:

- the investigator will stop collecting information about them; and
- they can ask the investigator to destroy any identifiable samples taken from them.

The investigator will ask withdrawn subjects to return unused trial medication, and to consent to a follow-up examination, to check that they have come to no harm as a result of taking part in the trial. Provided that subjects agree, they will undergo, at withdrawal from the trial, the standard medical examination and laboratory tests which they would have undergone had they completed the treatment regimen. The investigator will record in the CRF the results of the follow up examination of withdrawn subjects, if they give their consent for that.

For the extended treatment period, the following criteria apply:

Patients will be withdrawn from the study if they:

- have ALT or AST values >3 times the upper limit of normal;
- are found to have a prolonged QTc value (QTc ≥ 500 ms or QT prolongation with an increase of QTc > 60 ms compared to baseline);
- show any evidence of suspected drug-drug interaction; or
- experience an SAE or SUSAR which can reasonably be attributed to YF476.

If one of the criteria above is met in 2 or more subjects, dosing will be stopped in all subjects.

11 Treatments

11.1 Treatments administered

Patients will take 50 mg YF476 by mouth once daily for up to 12 weeks. Investigators have the option to increase the dose to 75 or 100 mg *once* daily after 6 weeks' treatment (at Visit 4). If a satisfactory response is seen with 50 mg YF476 *once* daily after 6 weeks for the first few patients, subsequent patients may be started on 25 mg YF476 *once* daily.

For the extended treatment period, patients will receive the same daily dose of YF476 as they did at the end of the initial 12 week period. The investigator will still have the option to increase the patient's dose to 75 or 100 mg once daily, or reduce it to 25 mg, depending on progress.

11.2 Labelling

HMR Pharmacy will label YF476 in accordance with GMP Annex 13. Each subject's treatment will be given a unique code number, traceable to the batch number.

11.3 Selection and timing of dose for each subject

Patients will take 50 mg YF476 by mouth with a glass of water with breakfast. If the dose is increased to 75 or 100 mg, that too will be taken with breakfast. On study visit days, patients will bring their container of YF476 to the clinic to take their dose.

11.4 Previous and concomitant treatment

Information on co-administered medicines is provided in Appendix 1. The investigator will include information about co-administered medicines in the letter to the patient's general practitioner.

Subjects must record all concomitant treatments in the diary card. Concomitant treatments will be reported in the case report form (CRF) along with their daily dosage, duration and reasons for administration. Subjects who have received any concomitant treatment may be withdrawn from the trial at the discretion of the investigator.

11.5 Assessment of compliance

Subjects will record in their diary card the date and time of each dose. At each clinic visit the diary card and YF476 container with be checked for compliance.

12 Main outcome measures

12.1 Efficacy

Efficacy will be assessed as follows:

- visual assessment of the number, size and distribution of gastric carcinoids at gastroscopy;
- histology and real-time PCR on biopsies for ECL-cell markers and biomarkers (such as CgA); and
- levels of fasting serum gastrin and plasma CgA, and trough and peak plasma YF476.

12.2 Safety and tolerability

Vital signs, results of physical examinations, any adverse events (see section 16), laboratory safety variables (see section 14), and ECG variables. Evidence of QTc prolongation will be sought.

13 Dietary and lifestyle restrictions

Subjects must make up to 12 visits to the clinic. Before each visit, patients must eat no food for 6 hours and before each gastroscopy visit they must also drink no fluids for 6 hours. They can eat and drink 30 min after gastroscopy.

Subjects must not take certain medicines before, and during, YF476 treatment (see Appendix 1). Pre-menopausal women who are at risk of pregnancy must use one of the recommended methods of contraception for the duration of YF476 dosing (see Section 10.2).

14 Procedures and observations

14.1 Visit 1: Screening for eligibility (part 1)

Patients will be asked to fast for 6 hours before this screening visit. The investigator will complete the following evaluation for each subject and enter the data from this visit, and all subsequent visits, on the appropriate page of the CRF:

- informed consent;
- letter to GP;
- medical history (including evidence for diagnosis of CAG, such as pernicious anaemia, achlorhydria, parietal cell antibody, intrinsic factor antibody, endoscopy and biopsy), medical examination, vital signs (heart rate and blood pressure);
- 12-lead ECG;
- *H. pylori* test (if status unknown);
- fasting serum gastrin, plasma CgA and serum for storage;
- laboratory analyses:
  - ♦ haematology: Hb, MCV, haematocrit, WBC and differential, and platelets;
  - ♦ biochemistry: urea, creatinine, total bilirubin, total protein, albumin, globulin, alkaline phosphatase, AST, ALT, gamma GT, glucose, cholesterol, triglycerides, potassium, sodium;
  - ♦ urinalysis: dipstick test: protein, blood, ketones, glucose, leukocyte esterase, specific gravity, nitrites, pH; microscopy if the result of the dipstick test for protein, blood, leukocyte esterase or nitrites is abnormal; urine pregnancy test only in women of child-bearing potential.

The investigator will tell patients to fast before gastroscopy at Visit 2.

14.2 Visit 2: Screening for eligibility (part 2), and start of YF476 treatment

Visit 2 will be within a maximum of 4 weeks after Visit 1. Patients will be asked to fast from food and fluids for 6 hours before this screening visit. The investigator will assess for eligibility all of the patient's results from the first screening visit. Eligible patients will:

- have fasted;
- have a pregnancy test if they are a woman of child bearing potential;
- undergo baseline gastroscopy and gastric biopsies; if the presence of gastric carcinoids is confirmed, the patient will be given the following until the next visit, which will be 3 weeks after Visit 2:

- ♦ container of YF476 capsules (25 mg); 2 capsules to be taken with breakfast, starting on the day after gastroscopy; and
- ♦ diary card, to record YF476 doses, any adverse events, and any concomitant medication.

It might be possible to combine Visits 1 and 2 for some patients.

The investigator will tell patients to fast before Visit 3, and to bring their container of YF476 capsules with them at the visit.

14.3 Visit 3: 3 weeks after starting YF476

The investigator will:

- check that the patient has fasted;
- collect the diary card, and issue a new one;
- check the diary card for compliance with treatment, and for adverse events and concomitant medication;
- collect YF476 container and assess compliance;
- record a 12-lead ECG, and blood pressure and heart rate;
- collect blood and urine for safety tests, including a pregnancy test for women of child-bearing potential;
- collect blood for assay of fasting levels of gastrin, CgA, YF476 (trough), and serum for storage;
- after taking the fasting blood sample, give the patient their dose of YF476, wait 1 h, and then collect blood for assay of peak concentration of YF476;
- issue a second container of YF476 capsules (25 mg); 2 capsules to be taken with breakfast as before; and
- remind the patient to fast before their gastroscopy on Visit 4.

14.4 Visit 4: 6 weeks after starting YF476

The investigator will:

- check that the patient has fasted;
- collect the diary card, and issue a new one;
- check the diary card for compliance with treatment, and for adverse events and concomitant medication;
- collect YF476 container and assess compliance;
- do a medical examination, including blood pressure and heart rate measurements, and 12-lead ECG;

- collect blood and urine for safety tests, including a pregnancy test for women of child-bearing potential.
- collect blood for assay of fasting levels of gastrin, CgA, YF476 (trough), and serum for storage;
- do a gastroscopy and take gastric biopsies, and proceed as follows:
  - ♦ if on visual inspection the gastric carcinoids have regressed completely: stop YF476, omit Visits 5 and 6, and proceed to Visit 7, providing all safety assessments prove satisfactory; **or**
  - ♦ if on visual inspection gastric carcinoids are still present: continue 50 mg YF476 *once* daily or consider increasing the dose to 75 or 100 mg *once* daily, issue a third container of YF476 capsules (25 mg); issue a new diary card;
  - ♦ after collecting the fasting blood sample and doing the gastroscopy, give the patient their dose of YF476, wait 1 h, and then collect blood for assay of peak YF476 concentration; and
  - ♦ instruct the patient about Visit 5.

14.5 Visit 5: 9 weeks after starting YF476

The investigator will:

- check that the patient has fasted;
- collect the diary card, and issue a new one;
- check the diary card for compliance with treatment, and for adverse events and concomitant medication;
- collect YF476 container and assess compliance;
- record a 12-lead ECG, and blood pressure and heart rate;
- collect blood and urine for safety tests, including a pregnancy test for women of child-bearing potential;
- collect blood for assay of fasting levels of gastrin, CgA, YF476 (trough), and serum for storage;
- after collecting the fasting blood sample, give the patient their dose of YF476, wait 1 h, and then collect blood for assay of peakYF476 concentration;
- issue a fourth container of YF476 capsules (25 mg); 2 capsules to be taken once daily with breakfast **or**, if the dose was increased at Visit 4, 3 or 4 capsules once daily with breakfast; and
- remind the patient to fast before their gastroscopy on Visit 6.

14.6 Visit 6: 12 weeks after starting YF476

The investigator will:

- check that the patient has fasted;
- collect the diary card;
- check the diary card for compliance with treatment, and for adverse events and concomitant medication;
- collect YF476 container and assess compliance;
- do a medical examination, including blood pressure and heart rate measurements, and 12-lead ECG;
- collect blood and urine for safety tests, including a pregnancy test for women of child-bearing potential;
- collect blood for assay of fasting levels of gastrin, CgA, YF476 (trough), and serum for storage;
- do a gastroscopy and take gastric biopsies, and proceed as follows:
    - ♦ if on visual inspection the gastric carcinoids have regressed completely, proceed to Visit 7; remind the patient to fast before their gastroscopy on Visit 7, **or**
    - ♦ if on visual inspection gastric carcinoids are still present, the patient leaves the trial.
- after collecting the fasting blood sample and doing the gastroscopy, give the patient their dose of YF476, wait 1 h, and then collect blood for assay of peak concentration of YF476.

14.7 Visit 7: 12 weeks after stopping YF476

Visit 7 will be a follow-up visit for patients who respond to YF476. The gastroscopy and blood sampling for fasting gastrin and CgA will be repeated.

Some patients will be eligible for extended treatment with YF476, as described below. To be eligible for further treatment, the patient must have benefitted from 12 weeks' treatment, and have tolerated YF476 well, and must give fully informed written consent.

14.8 Visit 8

The investigator will:

- tell the patient about the extended dosing period and ask him or her to read the information about the extended dosing and sign the consent form;

- do a gastroscopy and take gastric biopsies if the investigator thinks that a new baseline gastroscopy is necessary. If this is the case, the patient will have been told in advance and will be asked to fast for ≥6 h before the visit;
- collect blood for safety tests and for assay of fasting levels of gastrin and CgA if the investigator thinks that new baseline values are necessary;
- give the patient a container of YF476 capsules (25 mg) and instructions on how many to take daily with breakfast, starting the following day; and
- give the patient a diary card, to record YF476 doses, any adverse events, and any concomitant medication.

14.9 Visit 9: 12 weeks after starting extended YF476 dosing

The investigator will:

- check that the patient has fasted;
- collect the diary card, and issue a new one;
- check the diary card for compliance with treatment, and for adverse events and concomitant medication;
- collect YF476 container and assess compliance;
- record a 12-lead ECG, and blood pressure and heart rate;
- collect blood and urine for safety tests, including a pregnancy test for women of child-bearing potential;
- collect blood for assay of fasting levels of gastrin, CgA, YF476 (trough), and serum for storage;
- after taking the fasting blood sample, give the patient their dose of YF476, wait 1 h, and then collect blood for assay of peak concentration of YF476;
- issue a new container of YF476 capsules (25 mg) to be taken with breakfast as before; and remind the patient to fast before their gastroscopy on Visit 10.

14.10 Visit 10: 24 weeks after starting extended YF476 dosing

The investigator will repeat the procedures from visit 9 and:

- do a gastroscopy and take gastric biopsies, and proceed as follows:
  - ♦ if on visual inspection the gastric carcinoids have regressed completely: stop YF476, omit Visits 11 and 12, providing all safety assessments prove satisfactory; **or**
  - ♦ if on visual inspection gastric carcinoids are still present: continue treatment with YF476 once daily.

- after collecting the fasting blood sample and doing the gastroscopy, give the patient their dose of YF476, wait 1 h, and then collect blood for assay of peak YF476 concentration; and instruct the patient about Visit 11.

14.11 Visit 11: 36 weeks after starting extended YF476 dosing

The investigator will:

- check that the patient has fasted;
- collect the diary card, and issue a new one;
- check the diary card for compliance with treatment, and for adverse events and concomitant medication;
- collect YF476 container and assess compliance;
- record a 12-lead ECG, and blood pressure and heart rate;
- collect blood and urine for safety tests, including a pregnancy test for women of child-bearing potential;
- collect blood for assay of fasting levels of gastrin, CgA, YF476 (trough), and serum for storage;
- after taking the fasting blood sample, give the patient their dose of YF476, wait 1 h, and then collect blood for assay of peak concentration of YF476;
- issue a new container of YF476 capsules (25 mg) to be taken with breakfast as before; and remind the patient to fast before their gastroscopy on Visit 12.

14.12 Visit 12: 52 weeks after starting extended YF476 dosing

The investigator will repeat the procedures from Visit 8, do a gastroscopy and take gastric biopsies, but will not issue a new diary card or a new container of YF476. After collecting the fasting blood sample and doing the gastroscopy, the investigator will give the patient their dose of YF476, wait 1 h, and then collect blood for assay of peak YF476 concentration.

14.13 Flexibility of study days

It may not always be possible to carry out the gastroscopies on scheduled days. Therefore, if necessary, gastroscopy visits may be delayed or brought forward by up to 3 days. Other visits may be made up to 2 days before or after the scheduled day. Patients will be given sufficient YF476 to cover such delays.

14.14 Methods

Collection of samples for laboratory safety tests: Blood will be taken for haematology and biochemistry according to local procedures. Urine will be collected in 60 mL Universal containers. Sample handling materials will be provided by the investigator.

Processing of blood samples for laboratory safety tests: Blood samples for biochemistry and haematology will be processed according to local procedures and sent to Joint Pathology Services, Duncan Building, Royal Liverpool University Hospital.

Laboratory safety tests: The Royal Liverpool University Hospital Laboratory will do safety tests on blood samples according to local procedures.

The investigator will do safety tests on urine samples according to local procedures. If results of the tests are abnormal, the laboratory will do microscopy.

Pregnancy test: urine will be tested using a commercially available test kit.

Collection and processing of blood samples for serum gastrin and stored serum: Venous blood for serum gastrin assay and stored serum (2 × 2.5 mL) will be collected into polypropylene tubes and will be allowed to clot at room temperature for at least 20 min. After clotting, the tubes will be centrifuged at about 1500 G for 10 min at 4°C, and the serum transferred to smaller tubes in equal aliquots then stored at –20°C before shipment to the HMR Analytical Laboratory, Cumberland Avenue, Park Royal, London, NW10 7EW. Serum samples for gastrin assay and storage will be shipped once the patient has completed the study. All sample handling materials will be provided by the HMR Analytical Laboratory.

Collection and processing of plasma for CgA assays:

4 mL venous blood will be collected into an EDTA tube and centrifuged at about 1500 G for 10 minutes at 4°C. The plasma will be transferred into 2 equal aliquots in polypropylene tubes, and stored at –70°C, before shipment to the HMR Analytical Laboratory, Cumberland Avenue, Park Royal, London, NW10 7EW. Plasma samples for CgA assay will be shipped once the patient has completed the study. All sample handling materials will be provided by the HMR Analytical Laboratory.

Gastrin assay and plasma CgA assay: Serum gastrin and plasma CgA will be measured by the HMR Analytical Laboratory using commercially available enzyme linked immunoassays (ELISA).

Processing of blood samples for pharmacokinetic analysis: YF476 is light-sensitive, so blood samples (4 mL) will be taken into foil-wrapped or amber lithium heparin tubes, and immediately placed on ice. Samples will be centrifuged at about 1500 G for 10 minutes at 4°C. Plasma will be transferred to foil-wrapped or amber polypropylene storage tubes. Plasma samples will be stored at –20°C within 90

minutes after collection, and stored in darkness until dispatch. Samples will be sent to Analytical Services, St George's Hospital, for analysis of YF476.

Stored serum: serum will be stored by the HMR Analytical Laboratory for assay of trial-related markers only, which will be decided upon at a later date.

Gastroscopy: a gastroenterologist who is experienced and trained in the procedure will do the gastroscopies and biopsies. Subjects must have fasted from food and fluids for at least 6 hours before the procedure. Before the procedure, patients will choose whether they want to be sedated with intravenous midazolam, or have their throat sprayed with a local anaesthetic. After gastroscopy, patients will rest until they are fully recovered from the procedure. They may eat 30 min after the procedure.

During the procedure, the distribution, number and size of any gastric carcinoids will be assessed visually, and the tumors will be photographed. A pair of standard biopsy forceps will be used as an internal standard for size correction between images. Biopsies will be taken of the antrum, corpus and carcinoids for histology assessment and assessment of ECL cell markers and biomarkers (such as CgA).

Histologic evaluation: Dr Fiona Campbell, Liverpool, will do the histology. Histologic evaluation will include the following:

- haematoxylin and eosin (H&E) staining;
- immunostaining for neuroendocrine markers including CgA and synaptophysin; and
- histologic grading.

Samples will initially be read and reported through the standard pathology reporting system. At the termination of the study, all samples will be read by a single expert pathologist who is blinded to identifying patient information and to treatment status (pre-treatment, during-treatment or post-treatment).

ECL cell and biomarker evaluation: The Physiology Laboratory, Liverpool, will do the ECL-cell markers. Professor Andrea Varro, Liverpool, will do the biomarkers.

Blood pressure and heart rate will be measured using an automatic method. At screening, measurements will be made with subjects in the supine position; subjects will remain supine for at least 3 min before vital signs are measured.

Standard 12-lead ECGs will be recorded using an electrocardiograph. Each recording will be printed on a single A4 page at paper speed 25 mm/sec and calibrated to 1 cm/mV, or to 0.5 cm/mV if the amplitude of the QRS complex requires that. Recordings will be made with subjects in a supine position; subjects will remain supine for at least 10 min before the ECG is recorded.

Medical examinations will be done by a physician. The following sites will be examined: head, neck, ears, nose, throat, eyes, chest, lungs, heart, abdomen, skin, and

lymph nodes; and the following systems will be assessed: musculoskeletal and neurological.

H. pylori test: The test will be done, according to local standard operating procedures, only if the patient's *H. pylori* status is unknown.

Diary card: Patients will record daily in the diary card: adverse events, YF476 treatment, and any other medicines taken.

Adverse Events: Adverse events will be recorded in the patient's diary card. The investigator will also question patients about adverse events at each visit.

14.15 Total volume of blood removed

The total volume of blood taken from each subject in the trial will be up to 275 mL (including 'overage' for repeat safety tests if necessary).

14.16 Precautions

As YF476 is light-sensitive, special care is needed to prevent its degradation during the handling of blood and plasma samples. Containers must be lightproof, or be foil-wrapped and amber tinted if they are translucent. If that is not feasible, materials must be handled in darkened rooms, with light of a wavelength >450 nm and an intensity below 20 Lux.

15 Trial materials

15.1 Active pharmaceutical ingredient (API): YF476

Structural formula:

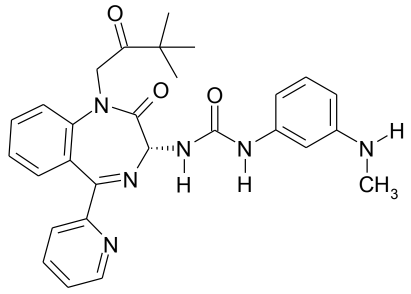

Chemical formula:	(R)-1-[2,3-dihydro-2-oxo-1-pivaloylmethyl-5-(2-pyridyl)-1 H -1,4-benzodiazepin-3-yl]-3-(3-methylaminophenyl)urea
Molecular formula:	$C_{28}H_{30}N_6O_3$
Molecular weight:	498.58
Description:	white to off-white / yellow crystals or crystalline powder
Purity:	≥ 99.0%
Melting point:	about 230°C (with decomposition)
Optical rotation:	$[\alpha]D^{20}$=+120–128°C (c= 0.5% in acetone)
Acid dissociation constant (pKa):	2.53, 4.59
***Hygroscopicity*:**	<0.3% at room temperature, and 93% relative humidity (7 days)
Stability:	Chemically stable for at least 9.5 years at room temperature, protected from light.

15.2 Formulated intermediate: spray-dried YF476 and HPMC

YF476 and HPMC (YF476:HPMC ratio 1:3.5) were dissolved in dichloromethane/isopropanol (7:2 v/v). The solution was spray-dried, and at all stages of production, the solution and resulting spray-dried powder were shielded from light. The formulated intermediate was characterised as follows.

Description: white to off-white powder

Structural configuration: amorphous by x-ray powder diffraction

YF476 purity: ≥ 95% of the theoretical purity (adjusted for water)

Tg: > 75°C

Water content: ≤ 7.5%

Residual dichloromethane: ≤ 2000 ppm

Residual isopropanol: ≤ 10000 ppm

Stability: based on the available data, and predictions from statistical analysis on the rate of change of purity and total related substances during storage at 2–8°C, spray-dried YF476, stored at 2–8°C, has been given a shelf-life of 4 years at 2–8°C.

15.3 Final formulation

The co-formulation of spray-dried YF476 and HPMC will be blended with starch, and placed into hard gelatine capsules by HMR Pharmacy. Each capsule will contain 25 mg YF476. The sponsor will provide a certificate of analysis for the test product, and any other documents and data required by the Qualified Person to release batches of investigational medicinal product (IMP).

15.3.1 Packaging and labelling

The trial medication will be packaged and labelled for each subject by the HMR Pharmacy, in accordance with The Rules Governing Medicinal Products in the European Union, Volume 4: Good Manufacturing Practice (GMP), and HMR's Manufacturing Authorisation for Investigational Medicinal Products [MIA(IMP)]. The IMP labels will include all the information required by Revision 1 of Annex 13 to GMP, as follows:

- name of the sponsor;
- pharmaceutical dosage form, route of administration, quantity of dose units, and name and strength of the medication;
- batch number and/or code number to identify contents and packaging operation;
- trial subject identification number;
- name of investigator;
- directions for use;
- 'clinical trial use only';
- trial reference code, allowing identification of the trial site and investigator;
- storage conditions;
- period of use (use-by date, expiry date, or re-test date, as applicable) in month/year format; and
- 'keep out of reach of children'.

15.3.2 Storage and accountability of IMP

The HMR Pharmacy will despatch the IMP to the investigator after ethical and regulatory approval of the trial have been obtained. The investigator will record and acknowledge in writing the receipt of all supplies of IMP.

The IMP will be stored securely at 2–8 ºC, accessible only to those individuals authorised by the investigator to dispense the IMP.

An inventory will be maintained, which will include the description and quantity of IMP received. A record will be kept of the IMP that is dispensed, to which subject, and the date and time of administration.

At the end of the trial, all unused IMP supplies will be returned to the sponsor.

16 Adverse events

16.1 Definitions of adverse events

Adverse event

Any untoward medical occurrence in a patient or clinical trial subject administered a medicinal product and which does not necessarily have a causal relationship with that treatment.

Adverse drug reaction

All untoward and unintended responses to an investigational medicinal product related to any dose administered.
Note that, according to the ICH Guideline for Good Clinical Practice (ICH GCP), a causal relationship between the medicinal product and the adverse event is at least a reasonable possibility, ie a relationship cannot be ruled out.

Unexpected adverse drug reaction

An adverse reaction, the nature or severity of which is not consistent with the applicable product information (eg Investigator's Brochure for an unauthorised investigational product, or summary of product characteristics for an authorised product).

Serious adverse event or serious adverse drug reaction

An adverse event or adverse drug reaction that is:

- fatal;
- life-threatening;
- requires or prolongs inpatient treatment;
- results in persistent or significant disability or incapacity; or
- is a congenital anomaly or birth defect.

Note:

- the term 'life-threatening' in the definition of 'serious' refers to an event or reaction in which the patient was at risk of death at the time of the event; it does not refer to an event or reaction which hypothetically might have caused death had it been more severe; and
- in accordance with the ICH Guideline on Clinical Safety Data Management: Definitions and Standards of Expedited Reporting, events or reactions that are not immediately life-threatening or may not result in death or hospitalisation, but might jeopardise the subject or require intervention to prevent one of the other outcomes listed above, should usually be considered serious.

Significant adverse event or reaction

An adverse event or reaction which is not serious, but is otherwise significant. The following will be considered significant:

- a marked haematological or other laboratory abnormality;
- an adverse event or reaction that leads to an intervention, including withdrawal of drug treatment, dose reduction or significant additional concomitant therapy; or
- any adverse event or reaction that the investigator considers to be significant.

16.2 Procedures for recording adverse events

Subjects will be carefully monitored for adverse events. The investigator or delegate will question the subjects about adverse events using a non-leading question, such as 'How are you feeling?' The investigator will also record adverse events reported spontaneously by the subjects. Clinically significant changes in the findings of physical examination, and clinically significant abnormalities in the results of objective tests (eg laboratory variables, x-ray, ECG) may also be recorded as adverse events. The investigator will use the following criteria when deciding whether to report an abnormal result as an adverse event.

1. The test result is associated with accompanying symptoms.
2. Results of additional diagnostic tests cause concern or necessitate medical intervention.
3. As a consequence of the test result, the dose administered to the subject is changed, the subject is withdrawn, or the subject is given concomitant treatment.
4. The investigator considers the result to constitute an adverse event.

If any of the above criteria are met, the investigator will report the result as an adverse event.

A record will be kept in the CRF of all adverse events as reported, whether believed to be related or unrelated to the treatment. The record will include the following.

- Clinical symptoms: a simple, brief description.
- Date and time of onset and end of clinical symptoms.
- Frequency: constant or intermittent.
- Severity. The following categories will be used:

  Mild: the adverse event does not interfere with the volunteer's daily routine, and does not require intervention; it causes slight discomfort.

Moderate: the adverse event interferes with some aspects of the volunteer's routine, or requires intervention, but is not damaging to health; it causes moderate discomfort.

Severe: the adverse event results in alteration, discomfort or disability which is clearly damaging to health.

- Relationship to treatment: The assessment of relationship of adverse events to the administration of IMP is a clinical decision based on all available information at the time of the completion of the case report form. The following categories will be used.

  Possibly related
  An event for which, after careful medical evaluation, a connection with trial medication cannot be ruled out with certainty. The event occurs after exposure to the test product. The event may occur at a reasonable time in relation to the time of administration of the IMP, but might also be attributable to a commonly occurring alternative cause. Alternatively, the event may not occur at a reasonable time in relation to the time of administration of the IMP, but not be attributable to an alternative cause.

  Unlikely to be related
  An event which occurs before exposure to the test product, or which does not occur at a reasonable time in relation to the time of administration of the IMP, and can be attributed to the patient's condition or a commonly occurring alternative cause. Alternatively, the event is unrelated to the trial, eg road traffic accident, unless it can be demonstrated that the treatment could have caused the event.

- Action taken: none, drug treatment, subject withdrawn, other (specified).
- Outcome: cleared, still present, or not known.

16.3 Procedures for dealing with serious adverse events

In the event of any serious adverse event which, in the investigator's opinion, justifies termination or modification of the trial, dosing will be stopped and the sponsor will be informed immediately (within 24 h of the investigator becoming aware of the event) by telephone or facsimile, as follows.

Telephone:	0800 h–1800 h:	020 8961 4130
	1800 h–0800 h:	020 7602 3119 or 07850 510 572
Fax:	(24 hours):	020 8961 8665

For all serious adverse events, the investigator will complete a serious adverse event form and provide it to the sponsor and the chief investigator, either directly or by facsimile, within 24 h of his becoming aware of the event.

The chief investigator will notify the main research ethics committee (REC) of serious adverse events that occur during this trial, if applicable, in accordance with the standard operating procedures issued by NRES.

The sponsor will notify the Medicines and Healthcare products Regulatory Agency (MHRA) of all suspected unexpected serious adverse reactions (SUSARs), and will be responsible for ensuring that the main REC is notified of SUSARs, if applicable. SUSARs that are fatal or life-threatening must be notified to the MHRA and REC within 7 days after the sponsor has learned of them. Other SUSARs must be reported to the REC and MHRA within 15 days after the sponsor has learned of them.

16.4 Procedures for handling withdrawals due to adverse events

The investigator will assess the reason for withdrawal as far as possible and will fully record the circumstances and medical details. Provided that subjects give written informed consent, they will undergo the standard medical examination and laboratory tests at withdrawal from the trial which they would have undergone had they completed it (see also Section 10.4).

17 Data management and quality assurance

The data will be securely stored by the investigator. After the CRFs have been signed off by the principal investigator, the original NCR copies will be sent to the Data Management department at HMR, where they will be securely stored.

Data will be double-entered into a clinical database management system (ClinPlus Version 3.3), which is based on SAS® Version 9.2. Edit checks and generation of queries will be done in ClinPlus. Tabulations and listings will be produced using validated, trial-specific SAS® programs.

Any laboratory value outside the normal range will be flagged with an ‘H’ if it is higher than the normal range, and with an ‘L’ if it is lower. If, during the course of a trial, the variable changes by more than a predetermined amount, that value will receive a flag ‘I’ if increased, or ‘D’ if decreased. Therefore, if during the course of the trial a value falls outside the normal laboratory range and alters by more than the predetermined amount, it will attract a double flag. Note that the predetermined change refers to a change from baseline (‘screening’) measurements.

Any laboratory variable that receives a double flag will be listed separately, along with the associated variables. For example, if the serum creatinine were abnormal, then urea and electrolytes will also be listed. All double-flagged variables will be listed in the investigator’s report together with a comment or explanation.

Adverse events will be coded using the version of the Medical Dictionary for Regulatory Activities (MedDRA) that is current when the database is locked.

Data will be checked by the HMR Quality Assurance (QA) Department. In addition, the HMR QA Department will audit the trial report; that audit will include checks to ensure that statistical output is correctly reproduced in the report.

18 Statistical methods

18.1 Statistical analyses

Statistical analysis will be done by HMR. A Statistical Analysis Plan will be prepared by the HMR Statistics and Data Management Department after completion of the final protocol. The statistical software package SAS® (SAS Institute, Cary, NC), version 9.2, will be used for all summaries and analyses.

Efficacy data

The objective of this pilot study is to estimate the therapeutic response rate in the patients who receive YF476. The primary endpoint of the clinical response is defined as a 25% reduction in the size or number of endoscopically evident type I gastric carcinoids or a reduction of 25% in the gastric ECL cell density. Secondary endpoints include safety and changes in levels of biochemical markers (eg gastrin, CgA), histologic grade and quantitative PCR for ECL cell-specific products.

All variables will be summarised by descriptive statistics as appropriate. The mean or median of the following variables will be expressed as a percentage change of the mean or median response (as appropriate) compared to the baseline period: number of CgA ir-cells per unit area of mucosa CgA_{muc} and volume density of CgA ir-cells ρ_{CgA},

The data during the treatment period will be compared with baseline using ANOVA, with visit as fixed effect and subject as random effect. If insufficient data are available, the treatment comparison using ANOVA will not be done. Variables and parameters will be transformed before analysis, as appropriate.

Data from all subjects who complete the study without major protocol deviations will be included in the analysis of efficacy. The primary aim of the trial is to find out if YF476 can cause regression of gastric carcinoid tumours.

Interobserver variability will be reported to both endoscopic and histologic interpretations. A blinded endoscopist, who is unaware of the patient history, dose of YF476, or timing of the endoscopic examination, will review a random sample of endoscopic images to report the size of endoscopic lesions. In addition, a blinded pathologist, who is unaware of the patient history, dose of YF476, or timing of biopsy specimen, will review a random sample of histologic images to report the histologic grade and ECL cell density of the biopsy sample. Kappa statistics will be calculated to report the degree of agreement between the study and blinded endoscopists and between the study and blinded pathologists.

Safety data

Data from all subjects who received any trial treatment will be included in the analysis of safety.

Numerical data will be summarised using means or medians, and other descriptive statistics, according to the type and distribution of the data. Adverse events (see Section 16) will be listed and relationship to treatment assessed.

Pharmacokinetic data

Plasma YF476 concentrations will be used to determine if there is a relationship between YF476 dose and plasma CgA concentration or serum gastrin concentration.

18.2 Determination of sample size

The trial is an exploratory, open, pilot trial, so a power calculation is inappropriate. Gastric carcinoids are a rare condition, so the protocol sets a limit of up to 10 patients.

The results will be reviewed regularly, and if the results are unfavourable, the study will be stopped. If the results are favourable, the sponsor will set up a larger trial, probably double-blind, randomised, and placebo-controlled in design, and with a fixed dose of YF476.

19 Ethical and regulatory requirements

The trial proposal will be reviewed by a recognised REC, and by the MHRA. The trial will not proceed unless the sponsor obtains from the MHRA a clinical trial authorisation (CTA), and the main REC approves the trial. The trial will not proceed until a site-specific assessment has been done by a REC and approval for the site has been given.

The trial will be done in compliance with EU Directives 2001/20/EC and 2005/28/EC, The Medicines for Human Use (Clinical Trials) Regulations 2004 and current amendments, the Declaration of Helsinki (South Africa Revision, 1996), GMP, the standard operating procedures issued by NRES for RECs in the UK, and Good Clinical Practice.

All patients must give written consent to participate in this trial. The trial-specific Information and Consent Form will be signed by the patient before any screening evaluation. Before giving consent, subjects must read the information sheet about the trial. They must also read the consent form. They will then discuss the trial with the investigator or his deputy and be given the opportunity to ask questions. The trial-specific Information and the Consent Form must have been approved by the main REC.

Each patient is free to withdraw from the trial at any time, without giving a reason. If a patient withdraws, the investigator will ask the patient to consent to a follow-up

examination. For withdrawn patients, the investigator will use a special Information and Consent Form. If the patient consents to the follow-up examination but asks the investigator to destroy all identifiable samples taken from the subject and/or not enter into the CRF results of the follow-up examination, the investigator will comply with the patient's requests.

The sponsor will ensure that the MHRA and the main REC are informed promptly of SUSARs (see Section 16.3), and that any new reports of SUSARs from other ongoing trials of the IMPs under investigation in this trial are notified to the MHRA, and to the main REC, if applicable. The sponsor will provide the investigator, the main REC and the MHRA with annual safety reports of each IMP under investigation, and listings of all suspected serious adverse reaction (SSAR) reports. If applicable, the sponsor will also provide the investigator, the main REC and the MHRA with 6-monthly safety reports, including listings of all SUSARs.

The investigator will promptly inform the sponsor and chief investigator of any SAE that occurs during this trial (see Section 16.3). The chief investigator will in turn inform the main REC, if applicable (see Section 16.3). The chief investigator will provide the main REC with annual progress reports of the trial, if it lasts longer than a year.

The investigator will report to the sponsor and chief investigator any protocol deviation that is, in his opinion, of clinical significance. The investigator will also inform the sponsor and chief investigator in the event of several deviations which, although of no clinical significance, cause inconvenience and/or discomfort to the volunteers. The chief investigator will in turn promptly inform the main REC.

The sponsor will notify the MHRA and main REC of any serious breach of Good Clinical Practice (for example, the investigator puts subjects' safety at risk, falsifies data, or persistently fails to comply with this protocol or Good Clinical Practice).

Within 90 days after the end of the trial, the sponsor will ensure that the main REC and the MHRA are notified that the trial has finished. If the trial is terminated prematurely, those reports will be made within 15 days after the end of the trial.

The sponsor will supply a summary report of the clinical trial to the MHRA and main REC within 1 year after the end of the trial.

Trial procedures will be subject to audits by the sponsor to ensure compliance with the protocol and applicable regulatory requirements.

20 Trial documentation

20.1 Protocol amendments

After the protocol has been approved by the main REC and the MHRA, no changes may be made without the agreement of both the investigator and the sponsor.

The MHRA and main REC do not need to approve any substantial change to the protocol that needs to be implemented urgently to avoid an immediate hazard to trial subjects. The sponsor will ensure that the MHRA and main REC are informed of urgent amendments in accordance with the detailed guidance to EU Directive 2001/20/EC and the standard operating procedures issued by NRES for NHS RECs.

All agreed protocol amendments will be recorded on a written agreement which will be signed and dated by the investigator and sponsor, and attached to the original protocol. The REC and/or MHRA must approve substantial amendments before they are implemented.

The following changes to the trial plan are permitted by this protocol, and will not be considered as amendments.

(a) Additional visits, for example to monitor blood pressure, will be arranged if there is a potential risk of a drug-drug interaction (see Appendix 1).

(b) Additional blood or urine samples may be taken for safety tests if required. The total volume of additional blood taken will not exceed 100 mL. Laboratory safety tests will be repeated, if the investigator has any safety concerns.

(c) Up to 3 additional visits to the clinic will be permitted, in the event of a technical failure and/or it extra safety tests are needed.

(d) Additional laboratory tests for ECL-cell markers, biomarkers and/or histology will be done on the gastric biopsies, if the results of other tests suggest that additional tests would be worthwhile.

(e) Stored serum will be used for laboratory tests not yet defined but related to this trial only.

(f) Visits 3 and 5 may be made up to 2 days before or after the scheduled day. Visits 4, 6 and 7 may be made up to 3 days before or after the scheduled day. Visits 9-12 may be up to 5 days before or after the scheduled day. Patients will be given sufficient YF476 to cover any delays. Therefore, up to 33 days extra YF476 may be taken by each patient, although every effort will be made to arrange patient’s visits on the scheduled days.

(g) It might not be possible to take a video of every gastroscopy. Photographs should suffice.

(h) If after the first few patients have completed the trial it is clear that increasing the dose at Visit 4 from 50 mg YF476 *once* daily to 75 or 100 mg *once* daily is necessary for a satisfactory response, and that the regimen is well tolerated, subsequent patients may be started on 75 or 100 mg *once* daily at their next visit.

(i) If after the first few patients a satisfactory response to the 50 mg YF476 dose *once* daily is seen at Visit 4, the dose may be reduced to 25 mg YF476 *once* daily, and subsequent patients may be started on this lower dose.

(j) Follow-up may be extended if:

  (i) a subject has an unresolved adverse event, which, in the opinion of the investigator, merits further follow-up; or

  (ii) new information becomes available that supports an extended follow-up period.

  The investigator will decide on the nature of the follow-up. For example, subjects may have a telephone follow-up at which they are asked about adverse events, or subjects may be asked to attend extra outpatient visits for additional monitoring of blood levels or effects of IMP, and for extra safety tests. The extra safety tests might include tests that are not described in this protocol. The investigator reserves the right, during or after the study, to do any extra safety tests that are in the best interest of the subjects. Those extra tests may or may not be described in this protocol.

(k) If the patient has not fasted for Visit 1, fasting safety tests of blood and urine may be done at Visit 2.

20.2 Case report forms

These will be designed and produced by HMR. The final version will be approved by the sponsor. All data will be entered legibly in black ink with a ball-point pen. If an error is made, the error will be crossed through with a single line in such a way that the original entry can still be read. The correct entry will then be clearly inserted and the alterations will be initialled and dated by the person making the alteration. Overwriting or use of correction fluid will not be permitted.

To preserve confidentiality, the CRFs will not bear the subject's name. The subject's initials, date of birth, and subject number will be used for identification.

It will be the responsibility of the investigator to ensure the accuracy of all data entered in the CRFs.

Source documents

Before the start of the study, the sponsor and investigator will sign an agreement listing the source documents to be used in this trial.

20.3 Reporting of results

HMR will prepare a draft report for discussion with the sponsor and investigators. The report will contain results and discussion of the trial, to which will be attached a full listing of all data recorded in the CRFs and summary tables of all important data.

Original copies of the CRFs will be kept, on behalf of the sponsor, by HMR.

21 Obligations of the sponsor and investigator

21.1 Monitoring and auditing

The trial will be monitored by the sponsor or delegate. All documents and data generated by the investigator will be audited by the sponsor or delegate.

All documents generated by HMR which form part of this trial, and the ensuing data, will be audited by the HMR Quality Assurance Department to assess compliance with the quality management system of HMR. That system incorporates the requirements of EU Directive 2001/20/EC, The Medicines for Human Use (Clinical Trials) Regulations 2004 (and current amendments), ICH GCP, EU Directive 2005/28/EC, GMP, and the standard operating procedures issued by NRES for RECs in the UK, and is compliant with ISO 9001:2000.

The sponsor may do a quality assurance audit, and regulatory authorities may inspect this study, at any time during or after the study. The sponsor and investigators agree to allow auditors and inspectors direct access to all relevant documents, and to allocate time to discuss findings with auditors or inspectors.

21.2 Compensation of patients

The sponsor agrees to abide by the ABPI clinical trial compensation guidelines (January 1991), and undertakes to compensate the subjects for injuries which are considered, on the balance of probabilities, to have arisen as a result of their participation in the trial.

21.3 Confidentiality

All personal details of the participating subjects and the results of the trial will be kept strictly confidential. Each subject's GP (or equivalent physician) will be informed of the nature and timing of the trial.

All unpublished documents including the protocol, the CRF, and the Investigator's Brochure are confidential. Those documents cannot be disclosed to a third party without the written consent of the sponsor. However, submission of those documents to a REC is expressly permitted.

The investigator agrees that the sponsor maintains the right to use the results of this trial, in their original form and/or in a global report, for submission to governmental and regulatory authorities of any country.

21.4 Publication

If the data merit, the investigators and the sponsor will discuss the preparation of a manuscript for publication in a peer-reviewed professional journal or an abstract for

presentation, oral or written, to a learned society or symposium. Either party may undertake the task but both must agree to the strategy before the work is started. Each party will allow the other 30 days to comment before any results are submitted for publication or presentation. Authorship should reflect work done by the investigators and personnel of the sponsor, in accordance with generally recognised principles of scientific collaboration.

21.5 Archiving

The investigator and sponsor will keep in a trial master file all the essential documents required by GCP. The sponsor or delegate will ensure that the sponsor's master file and original copies of CRFs will be archived in a secure place for at least 15 years. The investigator will ensure that the investigator's master file, and all data generated during the trial will be archived in a secure place for at least 15 years. All documents will be stored such that they are readily available for inspection at the request of the sponsor or a regulatory authority. Any transfer of ownership of the investigator's data or documents will be documented, and the sponsor will be informed.

22 Premature termination of the trial

The sponsor and investigator reserve the right to terminate this trial should serious or severe adverse events or any other safety issue occur during the trial. If the trial is terminated prematurely, the investigator will return all CRFs to the sponsor, and the sponsor or investigator, as appropriate, will provide a written statement of the reasons for termination. The sponsor will ensure that the MHRA and main REC are notified, as described in Section 19.

23 References

Aihara T, Fujishita T, Kanatani K, Furutani K, Nakamura E, Taketo M, Matsui M, Chen D, Okabe S. Impaired gastric secretion and lack of trophic responses to hypergastrinemia in M_3 muscarinic receptor knockout mice. Gastroenterology 2003; 125: 1774–1784.

Ahlman H, Kolby L, Lundell L, Olbe L, Wangberg B, Granerus G, Grimelius L, Nilsson O. Clinical management of gastric carcinoid tumors. Digestion 1994; 55 Suppl 3: 77–85.

Bakke I, Qvigstad G, Sandvik A, Waldum H. The CCK-2 receptor is located on the ECL cell, but not on the parietal cell. Scand J Gastroenterol 2001; 36: 1128–1133.

Beltinger J, Hildebrand P, Drewe J, Christ A, Hlobil K, Ritz M, D'Amato M, Rovati L, Beglinger C. Effects of spiroglumide, a gastrin receptor antagonist, on acid secretion in humans. European Journal of Clinical Investigation 1999; 29: 153-159

Black J. Reflections on some pilot trials of gastrin receptor blockade in pancreatic cancer. European Journal of Cancer 2009; 45:360-364

Black J, Kalindjian B. Gastrin agonists and antagonists. Pharmacol Toxicol 2002; 91: 275–281.

Borch K. Atrophic gastritis and gastric carcinoid tumours. Ann Med 1989; 21: 291–7.

Borch K, Ahren B, Ahlman H, Falkmer S, Granerus G, Grimelius L. Gastric carcinoids: biologic behaviour and prognosis after differentiated treatment in relation to type. Ann Surg 2005; 242: 64–73.

Borch K, Stridsberg M, Burman P, Rehfeld J. Source of chromogranin A and gastrin concentrations in circulation correlate with endocrine cell proliferation in type-A gastritis. Arch Surgery 1997; 32: 198–202.

Boyce M, Warrington S, Johnston A, Harris A. Effect on gastric pH of single doses of YF476, a new gastrin antagonist, compared with ranitidine and placebo. Br J Clin Pharmacol 2000a; 49: 493–494.

Boyce M, Warrington S, Johnston A, Harris A. Effect on gastric pH of repeated doses of YF476, a new gastrin antagonist, compared with omeprazole and placebo. Br J Clin Pharmacol 2000b; 50: 383–384.

Boyce M, Warrington S, Lewis Y, Nentwich H, Harris A. Adaptation to the antisecretory effect of YF476, a new gastrin antagonist, in healthy men. Br J Clin Pharmacol 2002; 53: 437P.

Boyce M, Warrington S, Nentwich H, Norris V, Hull R, Black J. Effect of repeated doses of YF476, a gastrin antagonist, on pentagastrin-induced changes in volume, H+ content and pH of gastric aspirate in healthy subjects. Br J Clin Pharmacol 2004; 57: 684.

Burkitt M, Pritchard D. Pathogenesis and management of gastric carcinoid tumours. Aliment Pharmacol Ther 2006; 24: 1305–1320.

Campana D, Nori F, Pezzilli R, Piscitelli L, Santini D, Brocchi E, Corinaldesi R, Tomassetti P. Gastric endocrine tumors type 1: treatment with long-acting somatostatin analogs. Endocrine-Related Cancer 2008; 15: 337–342.

Chau I, Cunningham D, Russell C, Norman A, Kurzawinski T, Harper P, Harrison P, Middleton G, Daniels F, Hickish T, Prendeville J, Ross P, Theis B, Hull R, Walker M, Shankley N, Kalindjian B, Murray G, Gillbanks A, Black J. Gastrazole (JB95008), a novel CCK2/gastrin receptor antagonist in the treatment of advanced pancreatic cancer: results from two randomised controlled trials. Br J Cancer 2006; 94: 1107–1115.

Chen D, Zhao C-M, Norlén P, Björkqvist M, Ding X-Q, Kitano M, Håkanson R. Effect of CCK-2 receptor blockade on rat stomach ECL cells. Cell Tissue Res 2000; 299: 81–95.

Chen D, Zhao C-M, Nylander A-G, Håkanson R. Time course of hypertrophic and ultrastructural responses of rat stomach ECL cells to sustained hypergastrinaemia. Cell Tissue Res 1996; 284: 55–63.

Cui G, Takaishi S, Ai W, Betz K, Florholmen J, Koh T, Houghton J, Pritchard D, Wang T. Gastrin-induced apoptosis contributes to carcinogenesis in the stomach. Lab Invest 2006; 86: 1037–1051.

D'Adda T, Keller G, Bordi C, Höfler H. Loss of heterozygosity in 11q13-14 regions in gastric neuroendocrine tumors not associated with multiple endocrine neoplasia type 1 syndrome. Lab Invest 1999; 79:671-677

Dakin G, Warner R, Pomp A, Salky B, Inabnet W. Presentation, treatment, and outcome of type I gastric carcinoid tumours. J Surg Oncol 2006; 93: 368–372.

DelValle J, Yamada T. Amino acids and amines stimulate gastrin release from canine antral G–cells via different pathways. J Clin Invest 1990; 85: 139–143.

Ding X-Q, Lindström E, Håkanson R. Evaluation of three novel CCK-B/gastrin receptor antagonists: a study of their effects on rat stomach ECL cell activity. Pharmacol Toxicol 1997; 81: 232–237.

Dockray G, Varro A, Dimaline R, Wang T. The gastrins: their production and biological activities. Ann Rev Physiol 2001; 63: 119–139.

Dressler G, Spence J, Bailey D. Pharmacokinetic-pharmacodynamic consequences of cytochrome P4503A4 inhibition. Clin Pharmacokinet 2000; 38: 41–57.

Eissele R, Patberg H, Koop H, Krack W, Lorenz W, McKnight A, Arnold R. Effect of gastrin receptor blockade on endocrine cells in rats during achlorhydria. Gastroenterology 1992; 103: 1596–1601.

Ellison E, Sparks J, Verducci J, Johnson J, Muscarella P, Bloomston M, Scott Melvin W. 50-year appraisal of gastrinoma: recommendations for staging and treatment. J Am Coll Sug 2006;202:897–905.

EU Clinical Trials Directive (2001/20/EC) & detailed guidance documents.

European Commission. Commission decision of 14 June 2007 relating to the designation under Regulation (EC) No 141/2000 of the European Parliament and of the Council of "(R)-1-[2,3-dihydro-2-oxo-1-pivaloylmethyl-5-(2-pyridyl)-1 *H* -1,4- benzo-diazepin-3yl]-3-(3-methylaminophenyl)urea" as an orphan medicinal product. Notification Pursuant to Article 254 of the EC treaty 14 Aug 2007.

Flockhart DA. Drug Interactions: Cytochrome P450 Drug Interaction Table. Indiana University School of Medicine (2007). http://medicine.iupui.edu/clinpharm/ddis/table.asp. Accessed January 2010.

Food and Drug Administration, Office of Orphan Products Development. Decision of 11 September 2009 relating to the designation under section 526 of the Federal Food, Drug and Cosmetic Act (21 U.S.C 360bb) of "(R)-1-[2,3-dihydro-2-oxo-1-pivaloylmethyl-5-(2-pyridyl)-1 *H* -1,4- benzo-diazepin-3yl]-3-(3-methylaminophenyl)urea" as an orphan drug for the treatment of gastric carcinoids.

Fossmark R, Johnsen G, Johanessen E, Waldum HL. Rebound acid hypersecretion after long-term inhibition of gastric acid secretion. Aliment Pharmacol Ther 2005; 21: 149–54.

Fox M, Forgacs I. Gastro-oesophageal reflux disease. BMJ 2006; 332: 88–93.

Fykse V, Sandvik A, Qvigstad G, Falkmer S, Syversen U, Waldum H. Treatment of ECL cell carcinoids with octreotide LAR. Scand J Gastroenterol 2004; 39: 621–628.

Fykse V, Sandvik A, Waldum H. One-year follow-up study of patients with enterochromaffin-like cell carcinoids after treatment with octreotide long-acting release. Scand J Gastroenterol 2005; 40: 1269–74.

Gillen D, McColl K. Problems associated with the clinical use of proton pump inhibitors. Pharmacol Toxicol 2001; 89: 281–286.

Gillen D, McColl K. Problems related to acid rebound and tachyphylaxis. Best Practice & Research Clinical Gastroenterology 2001; 15: 487–495.

Gillen D, Wirz A, Ardill J, McColl K. Rebound acid hyper secretion after omeprazole and its relationship to on-treatment acid suppression and *H pylori* status. Gastroenterology 1999; 116: 239–247.

Håkanson R, Chen K, Anderson K et al. The biology and physiology of the ECL cell. Yale J Biology and Medicine 1994; 67: 123–134.

Håkanson R, Ding X-Q, Norlén P, Chen D. Circulating pancreastatin is a marker for the ECL-cells of the rat stomach. Gastroenterology 1995; 108: 1445–1452.

Håkanson R, Ding X-Q, Norlén P, Lindström E. CCK2 receptor antagonists: pharmacological tools to study the gastrin-ECL cell-parietal cell axis. Regulatory Peptides 1999; 80: 1–12.

Håkanson R, Tielemans Y, Chen D, Andersson K, Mattsson H, Sundler F. Time-dependent changes in ECL-cell kinetics in stomach of hypergastrinemic rats. Gastroenterology 1993; 105: 15–21.

Hersey S, Sachs G. Gastric acid secretion. Physiol Rev 1995; 75: 155–189.

Hirschowitz B, Worthington J, Mohnen J, Haber M. Chromogranin A in patients with acid hypersecretion and/or hypergastrinaemia. Aliment Pharmacol Ther 2007; 26: 869–878.

HMR report 96-001. A double-blind, placebo-controlled study to assess the safety, tolerability and pharmacokinetics of single rising doses of YF476, a novel gastrin/CCK-B antagonist, in healthy male volunteers. 7 Oct 1997.

HMR report 96-016. YF476: effects of a single dose at 3 dose levels on 24-hour ambulatory gastric pH compared with placebo and ranitidine in healthy volunteers. 11 Mar 1998.

HMR report 96-018. YF476: effects of repeated dosing at 2 dose levels on 24-hour ambulatory gastric pH compared with placebo and omeprazole in healthy volunteers. 16 Jan 1998.

HMR report 97-010. A double-blind, placebo-controlled, parallel-group study of the effect of 5, 10 and 25 mg of YF476 daily for 14 days on 24-hour ambulatory gastric pH and plasma gastrin in healthy volunteers. 15 Aug 2002.

HMR report 01-022. Double-blind, placebo-controlled, single-dose, 5-way crossover study of the effect of YF476 (1, 5, 25 & 100 mg) on pentagastrin-induced gastric acid output in healthy volunteers. 19 Sep 2002.

HMR report 01-023. Open study of the effect of repeated doses of YF476 on pentagastrin-induced gastric acid output in healthy volunteers. 27 Feb 2003.

HMR study 07-012. A single-centre, open-label study in healthy subjects to investigate the potential pharmacokinetic interaction between YF476 and midazolam.

HMR study 07-013. A 2-way crossover study to assess the bioavailability of YF476 in healthy men and women in the fed and fasted states.

Hodgson N, Koniaris L, Livingstone A, Franceschi D. Gastric carcinoids: a temporal increase with proton pump introduction. Surg Endosc 2005; 19: 1610–1612.

International Conference on Harmonisation of technical requirements for registration of pharmaceuticals for human use: Harmonised Tripartite Guideline for Good Clinical Practice

Investigator’s Brochure for YF476.

Jensen R. Consequences of long-term proton pump blockade: insights from studies of patients with gastrinomas. Basic Clin Pharmacol Toxicol 2006; 98: 4–19.

Jensen R, Fraker D. Zollinger-Ellison syndrome. Advances in treatment of gastric hypersecretion and the gastrinoma. JAMA 1994; 271: 1429–1435.

Jansen J, Klinkenberg-Knol E, Meuwissen S, De Bruijne J, Festen H, Snel P, Luckers A, Biemond I, Lamers C. Effect of long-term treatment with omeprazole on serum gastrin and serum group A and C pepsinogens in patients with reflux esophagitis. Gastroenterology 1990; 99: 621–628.

Kaltsas G, Besser M, Grossman A. The diagnosis and medical management of advanced neuroendocrine tumours. Edocrine Rev 2004; 25: 458–511.

Kidd M, Modlin I, Black J, Boyce M, Culler M. A comparison of the effects of gastrin, somatostatin and dopamine receptor ligands on rat gastric enterochromaffin-like cell secretion and proliferation. Regul Pept 2007; 143: 109–117.

Kitano M, Norlén P, Ding X-Q, Nakamura S, Håkanson R. Long-lasting CCK2 receptor blockade after a single subcutaneous injection of YF476 or YM022. Brit J Pharmacol 2000; 130: 699–705.

Konagaya T, Bernsand M, Norlén P, Håkanson R. Mobilization of rat stomach ECL-cell histamine in response to short- or long-term treatment with omeprazole and/or YF476 studied by gastric mucosal microdialysis in conscious rats. Br J Pharmacol 2001; 133: 37–42.

Laine L, Ahnen D, McClain C, Solcia E, Walsh J. Review article: potential gastrointestinal effects of long-term acid suppression with proton pump inhibitors. Aliment Pharmacol Ther 2000; 14: 651–668.

Martinsen T, Kawase S, Hakanson R, Torp S, Fossmark R, Qvigstad G, Sandvik A, Waldum H. Spontaneous ECL cell carcinomas in cotton rats: natural course and prevention by a gastrin receptor antagonist. Carcinogenesis 2003; 24: 1887–96.

Lamberts R, Creutzfeldt W, Struber H, Brunner G, Solcia E. Long-term omeprazole therapy in peptic ulcer disease: gastrin, endocrine cell growth, and gastritis. Gastroenterology 1993; 104: 1356–70.

Laxen F, Sipponen P, Ihamaki T, Hakkiluoto A, Dortscheva Z. Gastric polyps; their morphological and endoscopical characteristics and relation to gastric carcinoma. Acta Pathol Microbiol Immunol Scand [A] 1982; 90: 221–228.

Ligumsky M, Lysy J, Siguencia G, Friedlander Y. Effect of long-term, continuous versus alternate-day omeprazole therapy on serum gastrin in patients treated for reflux oesophagitis. J Clin Gastroenterol 2001; 33: 32–35.

Ljubicic N, Kujundzic M, Roic G, Banic M, Cupic H, Doko M, Zovak M. Benign epithelial gastric polyps: frequency, location, and age and sex distribution. Coll Antropol. 2002; 26: 55–60.

McDonald I. CCK2 receptor antagonists. Exp Opin Ther Patents 2001; 11: 445–462.

Modlin I, Lye K, Kidd M. A five-decade analysis of 13,715 carcinoid tumours. Cancer 2003; 97: 934–959.

Modlin I, Lye K, Kidd M. A 50-year analysis of 562 gastric carcinoids: small tumour or larger problem? Ann J Gastroenterol 2004; 99: 23–32.

Murphy MG, Sytnik B, Kovacs TO, Mertz H, Ewanik D, Shingo S, Lin JH, Gertz BJ, Walsh JH. The gastrin-receptor antagonist L-365,260 inhibits stimulated acid secretion in humans. Clinical Pharmacology and Therapeutics 1993; 54: 533-539

Niklasson A, Lindstrom L, Simren M *et al*. Dyspepsia symptom development after discontinuation of proton pump inhibitor: clinical importance of acid-rebound. Gastroenterology 2008; 134 (Suppl 1).

Nishida A, Kobayashi-Uchida A, Akuzawa S, Takinami Y, Shishido T, Kamato T, Ito H,Yamano M, Yuki H, Nagakura Y *et al*. Gastrin receptor antagonist YM022 prevents

hypersecretion after long-term acid suppression. Am J Physiol Gastrointest Liver Physiol 1995; 269: 699–705.

Norton J, Doppman J, Jensen R. Curative resection in Zollinger-Ellison syndrome. Results of a 10-year prospective study. Ann Surg 1992; 215: 8–18.

Pellicano R, De Angelis C, Resegotti A, Rizzetto M. Zollinger-Ellison syndrome in 2006: concepts from a clinical point of view. Panminerva Med 2006; 48: 33–40.

Qvigstad G, Waldum H. Rebound hypersecretion after inhibition of gastric acid secretion. Basic Clin Pharmacol Toxicol 2004; 94: 202–208.

Qvigstad G, Falkmer S, Westre B, Waldum H. Clinical and histopathological tumour progression in ECL-cell carcinoids ("ECLomas"). APMIS 1999; 107: 1085–1092.

Peracchi M, Gebbia C, Basilisco G, Q uatrini M, Tarantino C, Vescarelli C, Massironi S, Conte D. Plasma chromogranin A in patients with autoimmune chronic atrophic gastrtitis, enterochromaffin-like cell lesions and gastric carcinoids. Eur J Endocrinol 2005; 152: 443–448.

Pregun I, Gergics P, Dabasi G, Igaz P, Racz K, Tulassy Z. Serum chromograin A reflects regression of metastatic carcinoid during prolonged octreotide treatment. Eur J Gastroenterol Hepatol 2009; 21: 476–477.

Rappel S, Altendorf-Hofmann A, Stolte M. Prognosis of gastric carcinoid tumours. Digestion 1995; 56 :455–462.

Redfern W, Carisson L, Davis A, Lynch W, Mackenzie I, Palethorpe S, Siegl P, Strang I, Sullivan A, Wallis R, Camm AJ, Hammond T. Relationship between preclinical electrophysiology, clinical QT interval prolongation and torsade de pointes for a broad range of drugs: evidence for a provisional safety margin in drug development. Cardiovasc Res 2003; 58: 32–45.

Redrup M, Leaf F, Miyashita A, Watanabe T, Higuchi S, Chasseaud L, Cheng K. Validation of a liquid chromatographic-tandem mass spectrometric method for the measurement of (R)-1-[2,3- dihydro-2-oxo-1-pivaloylmethyl-5-(2-pyridyl)-1H-1,4-benzodiazepin-3-yl]-3-(3methylaminophenyl)urea (YF476) in human plasma. J Chromatography 2002; 772: 317–325.

Reimer C, Sondergaard B, Hilsted L, Bytzer P. Proton-pump inhibitor therapy induces acid-related symptoms in healthy volunteers after withdrawal of therapy. Gastroenterology 2009; 137: 80–87.

Richards M, Gauger P, Thompson N, Giodano T. Regression of type II gastric carcinoids in multiple endocrine neoplasia type I patients with Zollinger-Ellison syndrome after surgical excision of all gastrinomas. World J Surg 2004; 28: 652–658

Richardson CT, Walsh JH, Hicks MI, Fordtran JS. Studies on the mechanisms of food-stimulated gastric acid secretion in normal human subjects. J Clin Invest 1976; 58: 623–631.

Rindi G, Bordi C, Rappel S, La Rosa S, Stolte M, Solcia E. Gastric carcinoids and neuroendocrine carcinomas: pathogenesis, pathology, and behavior. World J Surg 1996; 20: 168–172.

Rindi G, Luinetti O, Cornaggia M, Capella C, Solcia E. Three subtypes of gastric argyrophil carcinoid and the gastric neuroendocrine carcinoma: a clinicopathologic study. Gastroenterology 1993; 104: 994–1006.

Roy P, Venzon D, Shojamanesh H, Abou-Saif A, Peghini P, Doppman J, Gibril F, Jensen R. Zollinger-Ellison syndrome. Clinical presentation in 261 patients. Medicine 2000; 79: 379–411.

Sanduleanu S, De Bruïne A, Stridsberg M, Jonkers D, Biemond I, Hameeteman W, Lundqvist G, Stockbrügger R. Serum chromogranin A as a screening test for gastric ECL cell hyperplasia during acid-suppressive therapy. Eur J Clin Invest 2001; 31: 802–811.

Semple G, Ryder H, Rooker D, Batt A, Kendrick D, Szelke M, Ohta M, Satoh M, Nishida A, Akuzawa S, Miyata K. (3R)-N-(tert-Butylcarbonylmethyl)-2-3-dihydro-2-oxo-5-(2-pyridyl)-1H-1,4-benzodiazepin-3-yl)-N'-(3-methylamino)phenyl)urea (YF476): a potent and orally active gastrin/CCK-B antagonist. J Med Chem 1997; 40: 331–341.

Sepulveda A. 2006. eMedicine, atrophic gastritis. http://www.emedicine.com/med/topic851.htm#section~workup

Shah R. The significance of QT interval in drug development. Br J Clin Pharmacol 2002; 54: 188–202.

Standard operating procedures for Research Ethics Committees in the UK. National Research Ethics Service.

Syversen U, Jacobsen MB, O'Connor DT, Ronning K, Waldum HL. Immunoassays for measurement of chromogranin A and pancreastatin-like immunoreactivity in humans: correspondence in patients with neuroendocrine neoplasia. Neuropeptides 1994; 26: 201–206.

Takemoto Y, Yuki H, Nishida A, Ito H, Kobayashi-Uchida A, Takinami Y, Akuzawa S, Ohta M, Satoh M, Semple G, Miyata K. Effects of YF476, a potent and selective gastrin/CCK-B receptor antagonist, on gastric acid secretion in beagle dogs with gastric fistula. Arzneim Forsch 1998; 48: 403–407.

Takaishi S, Cui G, Frederick DM, Carlson JE, Houghton J, Varro A, Dockray GJ, Ge Z, Whary MT, Rogers AB, Fox JG, Wang TC. Synergistic inhibitory effects of gastrin and histamine receptor antagonists on Helicobacter-induced gastric cancer. Gastroenterology 2005; 128: 1965–83.

Takinami Y, Yuki H, Nishida S et al. YF476 is a new potent and selective gastrin/cholecystokinin-B receptor antagonist in vitro and in vivo. Aliment Pharmacol Ther 1997; 11: 113–120.

The Medicines for Human Use (Clinical Trials) Regulations 2004. Statutory instruments 2004, 1031 & amendments.

The Rules Governing Medicinal Products in the European Union, Volume 4 – Good Manufacturing Practice, incorporating Directive 2003/94/EC.

Tu S, Chi AL, Lim S, Cui G, Dubeykovskaya Z, Ai W, Fleming J, Takaishi S, Wang T. Gastrin regulates the TFF2 promoter through gastrin-responsive cis-acting elements and multiple signalling pathways. Am J Physiol Gastrointest Liver Physiol. 2007; 292: G1726–1737.

Waldum H, Arnestad J, Brenna E, Eide I, Syversen U, Sandvik A. Marked increase in gastric acid secretory capacity after omeprazole treatment. Gut 1996; 39: 649–653.

Waldum H, Brenna E, Sandvik A. Long-term safety of proton pump inhibitors: risks of gastric neoplasia and infections. Expert Opin Drug Saf 2002; 1: 29–38.

Watson S, Grabowska A, El-Zaatari, Takhar A. Gastrin – active participant or bystander in gastric carcinogenesis? Nature Reviews 2006; 6: 936–946.

Yamanouchi report. Effect of chronic treatment with YF476 on basal and pentagastrin-stimulated gastric acid secretion and serum gastrin levels in rats. Data supplied by Ferring, 5 Feb 1997.

Yamanouchi report. Plasma concentration of unchanged YF476 in rats given YF476 subcutaneously for 14 days. Data supplied by Ferring, 28 Jan 1997.

Zhao CM, Hakanson R, Chen D. Secretory organelles in ECL cells: effects of pharmacological blockade of the gastrin/CCK2 receptor versus its elimination by gene targeting. Inflammopharmacol 2005; 13: 75–82.

Appendix 1 Restricted medication

Cytochrome P450

YF476 inhibited cytochrome P450 enzyme CYP3A4/5 and CYP2C8 in human liver microsomes *in vitro*. It is reasonable to assume that YF476 is also a substrate for those enzymes. YF476 could, in theory, interact with medicines whose metabolism is critically dependent on them.

Many medicines are metabolised via CYP3A4/5 (Dressler *et al* 2000). Very few are metabolised via CYP2C8. Examples of the main types of medicine that are metabolised by CYP3A4/5 and CYP2C8 are listed below (for a full list and links to specific literature references, see http://medicine.iupui.edu/flockart/table.htm). Some substrates are not necessarily dependent on a single CYP enzyme for their metabolism.

CYP3A4/5 substrates

- benzodiazepines: diazepam, midazolam and triazolam;
- calcium channel inhibitors: amlodipine, diltiazem, felodipine, nifedipine, verapamil, nitrendipine and nisoldipine;
- HMG-CoA reductase inhibitors: atorvastatin, cerivastatin and lovastatin. NOT pravastatin, simvastatin and rosuvastatin;
- steroid 6β-OH: oestradiol, hydrocortisone and progesterone;
- ergot alkaloids: ergotamine and dihydro-ergotamine;
- immune modulators: cyclosporine, tacrolimus and sirolimus;
- HIV antivirals: indinavir, nelfinavir, ritonavir and saquinavir; and
- miscellaneous: such as domperidone, propranolol, risperidone, tamoxifen, salmeterol and sildenafil.

CYP3A4/5 inhibitors

- macrolide antibiotics: clarithromycin and erythromycin;
- azole antifungals: ketoconazole, fluconazole, itraconazole and voriconazole; and
- miscellaneous: dofetilide, sertindole and pimozide.

CYP3A4/5 inducers

- rifampicin, barbiturates, carbamazepine, phenytoin, glucocorticoids, pioglitazone and St John's wort.

CYP2C8 substrates

- cerivastatin and paclitaxel.

CYP2C8 inhibitors

- trimethoprim, glitazones and montelukast.

CYP2C8 inducers

- rifampicin.

Assessment and management of risk of potential drug-drug interactions

Ideally, during early clinical trials, YF476 should be tested in patients taking no other medicines, until we know more about its effect on the pharmacokinetics of co-administered medicines, and *vice versa*. However, that would restrict patient recruitment and deny some patients access to a potential medical treatment for their gastric carcinoids. Furthermore, many of the potential drug-drug interactions could be avoided or mitigated by modifying the dose of the co-administered medicine or by substituting another that is metabolised in a different way. Potential recruits for this trial will mostly be aged over 50 years, and therefore might well be taking one or more types of medicine.

CYP3A4/5 substrates

Results from the study of YF476 and human microsomes *in vitro* suggest that the main risk to trial patients from an interaction would come from co-administered medicines that are substrates for CYP3A4/5. Inhibition of CYP3A4/5 by YF476 might decrease their metabolism and increase their pharmacodynamic effects. So, we need to consider the various types of CYP3A4/5 substrate and their potential for interactions, and how those interactions might be managed.

- Steroid 6β-OH: Patients using a steroid contraceptive (oestradiol/progesterone) are already excluded from the trial. Moreover, most of the potential female recruits to the trial will probably be postmenopausal.
- HIV antivirals or immune modulators: Patients requiring these are unlikely to be among potential recruits, so they too will be excluded from the trial.
- Benzodiazepines: Possible side effects of decreased metabolism of benzodiazepines – such as sedation and dizziness – are unlikely to prove harmful, and moreover could be managed by reducing the dose of the benzodiazepine or stopping it, if necessary.
- Ergot alkaloids: These are usually taken as a single dose or short course for treatment of migraine. Any decrease in their metabolism is unlikely to prove harmful. If necessary, a triptan could be substituted for an ergot alkaloid.
- Calcium channel inhibitors: Patients taking one of these can continue to do so, providing their blood pressure and heart rhythm are monitored more closely and the dose adjusted, if necessary.
- HMG-CoA reductase inhibitors (‘statins’): Patients taking a statin that is metabolised via CYP3A4/5 should have their dose reduced, say by half, to mitigate the risk, albeit low, of rhabdomyolisis. Alternatively, a statin that is not

metabolised via CYP3A4/5 – such as pravastatin, simvastatin and rosuvastatin – could be substituted for one that is.

- Other types of CYP3A4/5 substrate: Any that pose a potential risk should be managed in a manner similar to the above types.

CYP2C8 substrates

YF476 might also decrease the metabolism of co-administered medicines that are substrates for CYP2C8, and thereby increase their pharmacodynamic effects. However, few medicines are metabolised via CYP2C8.

- Cerivastatin: This is another HMG-CoA reductase inhibitor and one of the few medicines that are metabolised via CYP2C8. The risk of an interaction could be mitigated by reducing the dose or replacing it with another statin, as appropriate.

Inhibitors or inducers of CYP3A4/5 and CYP2C8

If YF476 is a substrate for CYP3A4/5 or CYP2C8, *inducers* of those enzymes might increase the metabolism of YF476 and reduce its pharmacodynamic effect, but that would not be a safety matter. In contrast, *inhibitors* of those enzymes might decrease the metabolism of YF476 and increase its pharmacodynamic effect or toxicity.

YF476 has been well tolerated in phase 1 trials and there is a good toxicokinetic margin (Investigator's Brochure). But, the main concern about co-administration of an inhibitor and a substrate of CYP3A4/5 is prolongation of the QTc interval, which can lead to torsade de pointes (Shah 2000). However, YF476 has a low potential to prolong the QTc interval, and patients with QTc interval >450 msec will be excluded from the trial. Patients requiring a macrolide antibiotic or an azole antifungal should be excluded from the trial, since those medicines can prolong QTc interval and cause torsade de pointes when co-administered with certain CYP3A4/5 substrates – such as terfenadine, astemizole and cisapride – all of which have been withdrawn from the market.

Guidance for investigators

Investigators should always consider the possibility of a drug-drug interaction in any patient who develops an unexpected side effect while taking YF476 together with other treatments. Investigators who are unsure about the eligibility of a patient for the trial should discuss the matter with the chief investigator or the sponsor.
