# Supplementary material for: Netazepide, a Gastrin Receptor Antagonist, Normalises Tumour Biomarkers and Causes Regression of Type 1 Gastric Neuroendocrine Tumours in a Nonrandomised Trial of Patients with Chronic Atrophic Gastritis
Source: PLoS One. 2013 Oct 1;8(10):e76462. doi: 10.1371/journal.pone.0076462 (PMC3788129; doi:10.1371/journal.pone.0076462)
Supplement: Table S1 — Circulating biomarkers and endoscopic features. (DOCX) [file pone.0076462.s003.docx]

|  | Weeks | 0 | 3 | 6 | 9 | 12 | 24 |
| --- | --- | --- | --- | --- | --- | --- | --- |
| Serum Gastrin RIA (pmol/L) | Mean | 866.25 | 957.50 | 916.25 | 837.50 | 963.75 | 702.50 |
|  | SD | 385.87 | 356.68 | 366.77 | 318.62 | 509.85 | 159.12 |
|  | Range | 470-1750 | 540-1500 | 380-1550 | 490-1600 | 450-2000 | 500-970 |
| Serum Gastrin ELISA (pmol/L) | Mean | 554.81 | 617.63 | 537.38 | 574.63 | 613.15 | 518.31 |
|  | SD | 183.79 | 199.52 | 210.33 | 195.49 | 225.02 | 131.87 |
|  | Range | 331.5-953 | 365-890 | 211-896 | 335-904 | 293-953 | 367-742 |
| Plasma CgA (U/L) | Mean | 63.16 | 18.95 | 22.41 | 18.58 | 19.60 | 51.60 |
|  | SD | 31.04 | 9.90 | 18.17 | 10.93 | 11.96 | 22.64 |
|  | Range | 25.2-128 | 9.31-40.9 | 9.14-68.8 | 8.45-45.4 | 9.08-48.6 | 24.3-98 |
| Tumour number | Mean | 11.37 | - | 8.63 | - | 7.25 | 7.38 |
|  | SD | 7.36 | - | 5.22 | - | 3.49 | 4.06 |
|  | Range | 4-30 | - | 2-20 | - | 2-12 | 1-14 |
| Tumour size (mm) | Mean | 8 | - | 6.5 | - | 5.5 | 5.125 |
|  | SD | 3.46 | - | 3 | - | 2.96 | 3.26 |
|  | Range | 3-15 | - | 2-10 | - | 2-10 | 2-10 |
