# Supplementary material for: Netazepide, a Gastrin Receptor Antagonist, Normalises Tumour Biomarkers and Causes Regression of Type 1 Gastric Neuroendocrine Tumours in a Nonrandomised Trial of Patients with Chronic Atrophic Gastritis
Source: PLoS One. 2013 Oct 1;8(10):e76462. doi: 10.1371/journal.pone.0076462 (PMC3788129; doi:10.1371/journal.pone.0076462)
Supplement: Table S2 — Mucosal biomarkers. (DOCX) [file pone.0076462.s004.docx]

|  | Weeks | 0 | 6 | 12 | 24 |
| --- | --- | --- | --- | --- | --- |
| HDC relative abundance | Mean | 0.0478 | 0.0132 | 0.0150 | 0.0503 |
|  | SD | 0.0298 | 0.0082 | 0.0098 | 0.0351 |
|  | Range | 0.0092-0.0975 | 0.0040-0.0311 | 0.0024-0.0312 | 0.0201-0.1367 |
| MMP7 relative abundance | Mean | 0.1324 | 0.0752 | 0.0489 | 0.0855 |
|  | SD | 0.1501 | 0.0517 | 0.0262 | 0.0346 |
|  | Range | 0.0330-0.5100 | 0.0270-0.1771 | 0.0158-0.1081 | 0.0375-0.1475 |
| PAI1 relative abundance | Mean | 0.0080 | 0.0087 | 0.0060 | 0.0077 |
|  | SD | 0.0060 | 0.0042 | 0.0027 | 0.0061 |
|  | Range | 0.0023-0.0225 | 0.0020-0.0138 | 0.0023-0.0113 | 0.0028-0.0209 |
| PAI2 relative abundance | Mean | 0.0020 | 0.0046 | 0.0042 | 0.0038 |
|  | SD | 0.0018 | 0.0057 | 0.0050 | 0.0056 |
|  | Range | 0.0006-0.0056 | 0.0006-0.0189 | 0.0004-0.0148 | 0.0006-0.0180 |
| CgA relative abundance | Mean | 0.0328 | 0.0087 | 0.0106 | 0.0366 |
|  | SD | 0.0151 | 0.0028 | 0.0076 | 0.0202 |
|  | Range | 0.0152-0.0624 | 0.0052-0.0152 | 0.0032-0.0281 | 0.0078-0.0639 |
